# Microbiota-driven epigenetic programming of local immunity

**DOI:** 10.3389/fimmu.2026.1854085

**Published:** 2026-08-25

**Authors:** Su Jin Lim, Young Min Son

**Affiliations:** Department of Systems Biotechnology, Chung-Ang University, Anseong, Republic of Korea

**Keywords:** epigenetic programming, epigenome-microbiota axis, local immunity, metabolite, microbiome

## Abstract

The maintenance of local immunity requires stable tissue adaptation, implying that persistent environmental cues must continuously reinforce niche-specific immune programs. Microbiota-derived metabolites are increasingly recognized as such cues, acting not merely as metabolic byproducts but as epigenetic inputs that regulate histone modifications, DNA and histone methylation, and non-coding RNA networks. Through these epigenetic mechanisms, microbial metabolites reprogram key immune populations by promoting tolerogenic or immunostimulatory states, tuning effector and memory differentiation, and reshaping subset-specific functions in a tissue-dependent manner. Tissue-level studies have further shown that this microbiota–epigenome crosstalk influences barrier tolerance in the gut and skin, antiviral and inflammatory responses in the lung, metabolic and fibrogenic programs in the liver, and microglial immune tone in the brain. However, studies establishing a complete causal chain from a defined commensal source and metabolite availability to sensing or uptake pathway, chromatin-modifying enzyme or epigenetic mark, target gene locus, responding immune cell type, and validated immune outcome remain to be limited. Future studies should integrate causal microbial genetics, longitudinal metabolomics, cell-type-resolved epigenomics, and human validation to distinguish causal mechanisms from associative findings and enable safe tissue- and patient-specific therapeutic translation. This review therefore outlines the microbiota–metabolite–epigenome axis as a unifying framework for local immunity while emphasizing the mechanistic gaps that must be addressed to develop effective therapeutic strategies.

## 1 Introduction

The body is geographically compartmentalized into distinct anatomical and functional regions, each characterized by specialized tissue niches. Accordingly, each tissue must harbor immune cells with tissue-specific properties capable of rapidly countering pathogens that preferentially target that site. We refer to this ensemble of tissue-adapted immune programs as “local immunity”—the cumulative outcome of tissue adaptation, whereby immune cells integrate diverse local cues to respond appropriately to local challenges that threaten homeostasis (1–4). Local immunity is not autonomously generated by immune cells alone; it is continuously shaped by crosstalk with tissue-resident non-immune cells. At barrier surfaces, epithelial cells produce factors that initiate or suppress immune responses, while other tissue-resident structural cells provide niche-specific signals that influence immune-cell state and function. This continuous crosstalk is essential for maintaining local homeostasis (1, 3, 5).

How, then, do these cells acquire and maintain such tissue-specific programs within a given tissue niche? One integrative concept that explains this process is imprinting, in which initial challenges such as the microbiota, pathogens, and food antigens are encoded as changes in chromatin accessibility, histone modifications, DNA methylation, and non-coding RNA networks (3, 4). Collectively, these molecular changes constitute epigenetic modifications; however, these epigenetic modifications do not alter the underlying DNA sequence but durably reshape chromatin architecture and gene expression programs, thereby stabilizing tissue-specific functional states in immune and barrier cells (6, 7).

Through this imprinting process, tissue-specific immune and barrier cells integrate persistent local cues, including cytokines, microbiota, and associated metabolites, alongside oxygen tension and mechanical forces, to establish stable transcriptional and epigenetic states. These durable states, in turn, allow these cells to mount faster and more appropriately tune responses to diverse forms of tissue damage within that niche (8–10).

Among these local cues, microbiota, a complex community of microorganisms such as bacteria, fungi, protozoa, and viruses, is distinctive (11). Unlike other local signals that are induced or markedly amplified only during infection, injury, or inflammation, microbial communities are stably established within the host and have coexisted and coevolved with the associated host organisms for millions of years in largely mutualistic relationships (12, 13).

Therefore, tissue-specific immune and barrier cells, which reside within the same niche for prolonged periods, are constantly exposed to microbiota-derived products and metabolites released by the gut and other organs. This lifelong exposure is expected to exert particularly strong and durable effects on the transcriptional and epigenetic programs of these cells, leaving lasting imprints on local immunity (8, 9, 14).

Thus, this review aims to outline a mechanistic framework for the microbiota–metabolite–epigenome axis in local immunity by describing the processes through which microbial metabolites function as epigenetic cues, the mechanisms through which epigenetic machinery in tissue-specific immune and barrier cells interprets these signals, and how microbiota-driven trained immunity and epigenetic memory emerge from these interactions. Then, this review examines tissue-specific examples of microbiota–epigenome–immunity crosstalk, highlighting local immunity within different niches.

## 2 The microbiota–metabolite–epigenome axis in local immunity

### 2.1 Microbial metabolites as epigenetic cues

Microbiota-derived metabolites influence multiple layers of epigenetic regulation, from histone modifications and DNA methylation to non-coding RNA (ncRNA) networks. A critical first step in understanding this axis is to define the molecular mechanisms through which these metabolites are deployed as epigenetic cues and subsequently interpreted by immune cells (**Table 1**).

**Table 1:** Epigenetic modifications induced by microbiota-derived metabolites and related epigenetic inputs.

Epigenetic modification	Mechanism	Input (Category)	Target	Epigenetic mark	Ref.
Histone Acetylation	Substrate-driven	Acetate (i)	ACSS2	↑ histone acetylation	(16–18)
		Butyrate (i)	Direct metabolic conversion to Acetyl-CoA		(19, 20)
		Polyamine metabolism (iv)	TCA cycle rewiring → altered citrate/acetyl-CoA availability	Altered histone acetylation	(21)
	Enzyme-driven	Competitive inhibition of Class I/II HDACs → ↓deacetylation	Endogenous Class I HDAC inhibition → ↓ deacetylation	↑ histone acetylation	(22, 23)
		β-hydroxybutyrate (iv)			(24)
		Propionate/Butyrate (i)	p300 auto-acylation → ↑ HAT activity		(26)
		Palmitate cooperated with LPS (iii, iv)	↑p300 recruitment and RNA polymerase II binding at the IL6 promoter ; response reduced by HAT inhibition		(27)
		Inositol phosphates (iv)	Class I HDAC-corepressor stabilization	Maintained deacetylation (↓ acetylation)	(25)
Histone/ DNA Methylation	Substrate- driven	Folate, Vitamin B12, Choline. Betaine (i, iv)	One-carbon metabolism → SAM/SAH	↑ methylation potential (DNA + histone)	(29, 30, 32)
		SCFAs (i)	DNMT/HMT programs (context-dependent)	Locus-specific DNA methylation + H3K4/H3K27 methylation changes	(37–39)
	Enzyme- driven	Bile acids (ii)	DNMT3A + HMT (G9a) recruitment/retargeting	Reshaped local DNA/histone methylation landscapes	(40)
		Tryptophan metabolites (i)	AhR-linked regulation of HMTs + ncRNA-associated regulation	Locus-specific DNA/histone methylation remodeling	(42)
Histone Crotonylation	Enzyme-driven	Crotonate (iv)	↑ crotonyl-CoA → p300-catalyzed crotonylation	↑ Kcr at active promoters/enhancers	(43)
		SCFAs (i)	HDACs as decrotonylases (Class I-III) inhibition → ↓ decrotonylation	↑ Kcr (e.g., epithelial cells)	(44)
Non-coding RNAs	Substrate-driven	Vitamin B12, Folate (i, iv)	↑ methyl donor → ↑ miRNA expression	↑ miRNAs; downstream target repression (context-dependent)	(51)
	Enzyme-driven	Butyrate (i)	HDAC inhibition → miRNA induction		(47, 50)

This table summarizes representative inputs according to the type of epigenetic modification, mechanism of action, molecular target, and resulting epigenetic mark. Inputs are classified as follows: (i) microbiota-derived metabolites; (ii) microbiota-modified host metabolites; (iii) microbial products or structural components; and (iv) host-derived, dietary, pharmacological, or experimentally administered mechanistic analogues.

#### 2.1.1 Histone acetylation

Histone acetylation is a well-established epigenetic modification in which an acetyl group from acetyl-CoA is transferred to specific lysine residues on histone tails by histone acetyltransferases (HATs). This generally promotes an open chromatin configuration and increased transcriptional activity. Histone acetylation is dynamically regulated by HATs, which utilize acetyl-CoA as a substrate, and by histone deacetylases (HDACs) and sirtuins, which remove these marks (15).

Microbial metabolites modulate this balance through two principal mechanisms: (i) altering cellular acetyl-CoA availability for acetylation reactions, and (ii) directly affecting the activity or recruitment of acetylation/deacetylation enzymes. Among these, short chain fatty acids (SCFAs), including acetate, propionate, and butyrate, are the best characterized microbial metabolites that regulate histone acetylation through both mechanisms (8).

Through substrate-driven effects (mechanism i), metabolites such as acetate sustain histone acetylation by replenishing the acetyl-CoA pool. Indeed, acetate can be recaptured by nuclear-cytosolic acetyl-CoA synthetase 2 (ACSS2), preserving local acetyl-CoA availability under stress conditions (16). In glucose-restricted CD8+ T cells, acetate enhances histone acetylation and *IFNG* transcription in an ACSS2-dependent manner (17). This role may be particularly important in settings of mitochondrial dysfunction, such as cancer and chronic infection, where acetate serves as an alternative carbon source to mitochondrial citrate to support acetyl-CoA production and effector-associated gene regulation (18).

Similarly, butyrate contributes to histone acetylation through the metabolic conversion of butyrate to acetyl-CoA and, in some contexts, by increasing acetyl-CoA abundance together with H3K9 acetylation (H3K9ac), consistent with a dual mode of action involving both carbon supply and chromatin regulation (19, 20).

In contrast to acetate and butyrate, which can more directly contribute to acetyl-CoA supply, polyamine metabolism may also regulate histone acetylation indirectly by rewiring the TCA cycle. Because the TCA cycle is closely linked to citrate and acetyl-CoA availability, this metabolic rewiring can alter acetylation-related chromatin states. In CD4+ T cells, disruption of polyamine synthesis or hypusine formation rewires TCA cycle metabolism, leading to altered histone acetylation (21).

Through enzyme-directed effects (mechanism ii), SCFAs, particularly butyrate, directly modulate acetylation and deacetylation enzymes. Mechanistically, butyrate competitively inhibits class I and II HDACs by mimicking the associated acetyl-lysine substrate and binding the Zn²^+^ catalytic site, thereby blocking deacetylation (22, 23). In parallel, β-hydroxybutyrate has been shown to function as an endogenous class I HDAC inhibitor, suppressing deacetylation and promoting histone acetylation and transcriptional reprogramming (24). However, not all metabolites suppress deacetylation: Distinct from general SCFAs, inositol phosphates act as structural cofactors that bind to class I HDAC–corepressor complexes and stabilize the subsequent deacetylase activity (25). In addition to HDAC inhibition, SCFAs can directly enhance HAT activity. Indeed, propionate and butyrate are converted intracellularly into propionyl-CoA and butyryl-CoA, respectively, thereby inducing auto-acylation of the p300 acetyltransferase and enhancing the associated activity independently of substrate availability (26). Together, these findings establish microbiota-derived metabolites as dual epigenetic regulators of histone acetylation, acting through both metabolic substrate supply and direct enzymatic control.

Although palmitate is not typically classified as a microbiota-derived metabolite, this study provides a useful mechanistic example of how a metabolic substrate can cooperate with microbial component–driven signaling to promote acetylation-dependent chromatin remodeling. In human monocytes, palmitate potentiates the LPS response by increasing H3K9/H3K18 acetylation at the IL6 promoter, enhancing p300 recruitment, and promoting RNA polymerase II binding, and this response was reduced by HAT inhibition. In contrast, HDAC inhibition with trichostatin A or sodium butyrate mimicked the effect of palmitate. Together, these results suggest that both increased acetyltransferase activity and reduced deacetylation shift the acetylation balance toward promoter hyperacetylation and transcription-permissive chromatin (27).

In a related example focusing on microbial structural component–driven signaling, lipopolysaccharide (LPS), a major component of the outer membrane of Gram-negative bacteria, cooperated with IFN-γ to enhance CCL2 expression in monocytic cells. IFN-γ priming increased H3K27 acetylation at the proximal CCL2 promoter, while HAT inhibition with anacardic acid attenuated CCL2 induction and HDAC inhibition with trichostatin A enhanced the response to LPS, supporting an acetylation-dependent mechanism (28). Together, these examples indicate that microbiota-associated regulation of histone acetylation is not limited to microbial metabolites but can also involve microbial structural components that cooperate with host inflammatory cues to promote locus-specific chromatin remodeling.

#### 2.1.2 Histone and DNA methylation

Histone and DNA methylation share S-adenosylmethionine (SAM) as a universal methyl donor. Meanwhile, the gut microbiota influences these marks via at least two metabolite-dependent routes: (i) indirectly, by shaping host one-carbon metabolism and methyl-donor availability, and (ii) directly, by modulating the activity or locus-specific recruitment of DNA and histone methyltransferase/demethylase complexes (29, 30).

Gut microbes synthesize or metabolize folate, vitamin B12, choline, and betaine through indirect mechanisms, thereby supporting one-carbon flux and SAM production for both DNA and histone methylation. Since these methyl-donor metabolites are readily obtained through diet, this axis provides a practical route by which nutrition can tune methylation capacity (29, 30). Consistent with this, commensals such as *Lactobacillus* and *Bifidobacterium*, which are major folate producers in the colon, have been linked to host methylation potential. The metabolites in these commensals may support one-carbon metabolism and optimize the SAM/S-adenosylhomocysteine (SAH) ratio, which reflects the balance between methyl-donor availability and SAH-mediated inhibition of methyltransferases. This, in turn, promotes SAM-dependent methylation reactions and indirectly enhances DNA methyltransferase (DNMT) activity (29, 31).

Similarly, methyl-donor availability can shape histone methylation states, including H3K4 and H3K27 methylation, through diet-derived one-carbon metabolites such as choline, betaine, and methionine, as well as through DNA methylation (29, 32). Notably, this remodeling has been observed not only in the intestinal mucosa but also in the fetal liver and brain, underscoring the capacity of microbiota-associated one-carbon precursors to tune epigenetic programming at distal sites (32–34).

Comparatively, several microbial metabolites reprogram histone and DNA methylation through more direct mechanisms by modulating the expression, stability, or chromatin recruitment of methylation enzymes and their regulators, thereby reshaping locus-specific methylation landscapes without requiring global shifts in SAM availability (35, 36).

Although butyrate and propionate are best known as endogenous HDAC inhibitors, accumulating evidence indicates that SCFAs can also reduce the expression of DNA and histone methyltransferases, thereby altering methylation states in ways that may help alleviate pathological conditions such as cancer and obesity (37–39).

Bile acids, whose composition and signaling are shaped by the gut microbiota, are also regulated through direct methylation-dependent mechanisms. In particular, farnesoid X receptor (FXR), a bile acid-sensing nuclear receptor that maintains bile acid homeostasis, engages methyltransferase-containing chromatin-modifying complexes such as G9a and the activating signal cointegrator-2-containing coactivator complex (ASCOM). Notably, this pathway regulates the transcription of genes involved in bile acid biosynthesis and transport, including CYP7A1, NTCP, BSEP, and MRP2 (40), by inducing the small heterodimer partner (SHP) and, in cooperation with HDACs.

Microbiota-derived tryptophan metabolites enhance aryl hydrocarbon receptor (AhR) signaling, and AhR has been linked to methylation remodeling via altered expression and targeting of methylation regulators; examples include increased expression of specific histone methyltransferases (e.g., SUV39H1) and ncRNA-associated regulation (e.g., MALAT1–EZH2), collectively influencing locus-specific DNA and histone methylation (41, 42).

Overall, these observations indicate that microbiota-derived metabolites modulate locus-specific histone and DNA methylation, providing a mechanistic link between the local metabolic milieu and host epigenetic regulation.

#### 2.1.3 Histone crotonylation and non-coding RNAs

In addition to canonical acetylation and methylation, short-chain acylation marks and ncRNAs, such as microRNAs (miRNAs) that bind mRNA and promote degradation, further diversify the ways in which microbiota-derived metabolites interface with the host epigenome.

Histone lysine crotonylation (Kcr) is a short-chain lysine acylation mark derived from crotonyl-CoA and is commonly enriched at active promoters and enhancers, where Kcr is associated with transcriptional activation (43). The abundance of Kcr is largely determined by intracellular crotonyl-CoA availability and can be elevated by crotonate supplementation. Notably, p300, a canonical histone acetyltransferase, also functions as a crotonyltransferase, and in some contexts, histone crotonylation exerts stronger transcriptional effects than histone acetylation (43).

Mechanistically, microbiota-associated regulation of Kcr is closely linked to the histone acetylation axis, because class I HDACs act as both deacetylases and major decrotonylases, making HDAC inhibition a shared regulatory point for multiple lysine acyl marks (44). Interestingly, the same SCFA–HDAC axis may also alter ncRNA networks, as HDAC inhibition can relieve repression at specific ncRNA loci, thereby reshaping downstream post-transcriptional regulation (45).

In colorectal cancer (CRC) cells, butyrate-associated chromatin modulation has been linked to an anti-proliferative ncRNA circuit in which p57 is upregulated alongside suppression of c-Myc and the oncogenic miR-17–92 cluster, with HDAC3 implicated in CRC-associated epigenetic programming (46–48).

This HDAC-driven regulation of ncRNA expression is even more clearly illustrated in B cells. Valproic acid and butyrate, both HDAC inhibitors, enhance histone acetylation at host genes of microRNAs that target AID and Blimp-1, essential regulators of antibody responses. This promotes miRNA-mediated repression of AID and Blimp-1 expression, thereby suppressing antibody diversification and plasma cell differentiation. Thus, both protective and autoantibody responses are modulated, contributing to prolonged survival in lupus models (49, 50).

In addition to histone acetylation-based control, methylation-dependent regulation of ncRNA expression has been linked to metabolic dysfunction. Vitamin B12 and folate supplementation can modulate miR-21 expression through methylation; since miR-21 directly targets type 2 diabetes candidate genes such as *FTO*, *TCF7L2*, *CREBBP*/*CBP*, and *SIRT1*, this mechanism provides an epigenetic explanation for the interaction between vitamin B12 deficiency and type 2 diabetes (51).

Together, these observations identify Kcr and ncRNA programs as metabolite-responsive epigenetic layers that can be co-tuned by microbiota-derived metabolites through HDAC- and DNA methylation-dependent chromatin mechanisms, with broad relevance to cancer, inflammation, and metabolic disease.

### 2.2 Epigenetic machinery in local immune cells

**Figure 1:**
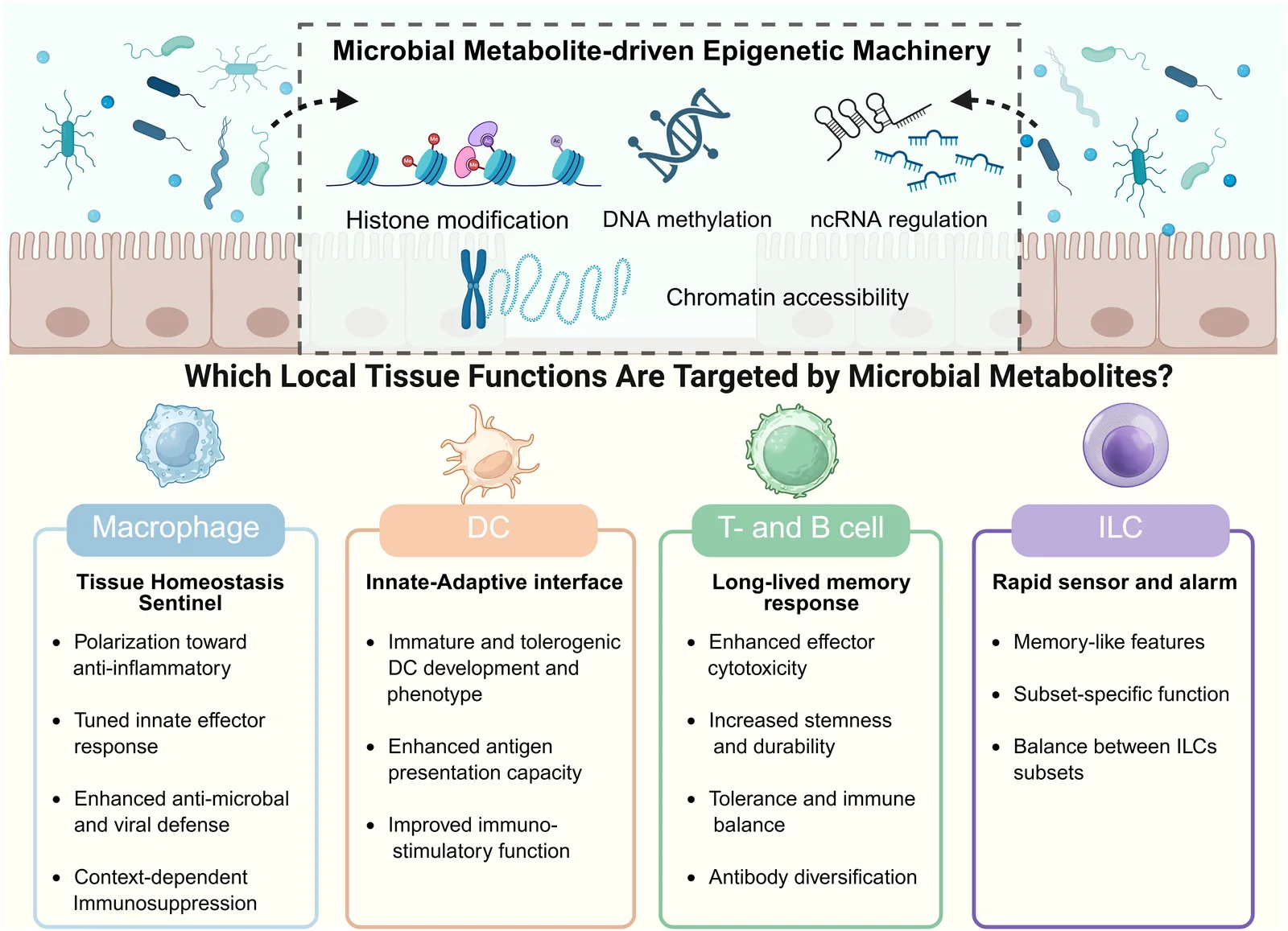
Epigenetic machinery in local immune cells. Schematic diagram illustrating how microbiota-derived metabolites engage multiple epigenetic mechanisms, including histone modification, DNA methylation, ncRNA regulation, and chromatin accessibility, to shape local immune cell states and functions. Through this epigenetic programming, macrophages are tuned to maintain tissue homeostasis, dendritic cells (DCs) acquire context-dependent antigen-presenting functions that promote innate–adaptive crosstalk, T and B cells develop enhanced memory durability and diversification, and innate lymphoid cells (ILCs) mediate rapid sensor and alarmin responses, thereby contributing to tissue-specific immune adaptation. Abbreviations: DC, dendritic cell; ILC, innate lymphoid cell; ncRNA, non-coding RNA.

A key question remains: how are these epigenetic layers interpreted by immune cells to generate the tissue-specific functions required within each niche? In particular, an important question is which tissue-specific immune functions are targeted by microbial metabolites and how these cues translate into the functional outputs required for local immunity (**Figure 1**).

#### 2.2.1 Macrophages

Macrophages orchestrate both innate immune defense and tissue homeostasis, exhibiting remarkable plasticity shaped by the associated developmental origins and local microenvironmental cues (52, 53). Notably, this plasticity supports tissue-specific epigenetic reprogramming and confers distinct functional identities (53). Microbiota-derived metabolites predominantly shape anti-inflammatory and homeostatic macrophage functions through epigenetic reprogramming, with SCFAs, particularly butyrate, emerging as central regulators of the transcriptional programs of alternatively activated (M2)-like macrophages (54, 55). Butyrate-driven HDAC inhibition consistently stabilizes pro-resolving macrophage phenotypes across diverse disease contexts. For example, in non-alcoholic steatohepatitis (NASH), sodium butyrate promotes NF-κB p65 acetylation and an M2-like pro-healing transcriptional program while depleting proinflammatory macrophages, collectively alleviating hepatic inflammation (54).

In addition to histone modifications, ncRNA networks mediate microbial metabolite signals that modulate macrophage polarization programs. *Clostridium butyricum*, a butyrate-producing bacterium, and the associated metabolite modulate miR-146a to drive M2 polarization via the SOCS7–JAK2–STAT3 axis in gouty arthritis (56). Similarly, an HDAC1/miR-155 axis mediates the anti-inflammatory effect of butyrate in alcoholic liver disease (57).

SCFA-mediated epigenetic remodeling extends beyond the canonical induction of M2 markers to context-dependent tuning of diverse macrophage effector functions relevant to innate immunity. In intestinal macrophages, n-butyrate selectively suppresses lipopolysaccharide (LPS)-induced nitric oxide, IL-6, and IL-12 through HDAC inhibition, independent of G protein-coupled receptor (GPCR) signaling, thereby establishing a homeostatic hyporesponsive state at barrier surfaces (58). Similarly, during bacterial sensing, butyrate and propionate suppress NF-κB activation, IFN-β induction, and STAT1 phosphorylation, thereby restraining excessive nitric oxide production. These effects are associated with HDAC inhibition and modulation of PRR- and IFN-associated inflammatory tone (59).

In addition to SCFA-mediated HDAC regulation in macrophages, studies in monocytic cells provide additional mechanistic insight into how locus-specific histone acetylation can cooperate with inflammatory transcription factor recruitment. In monocytic cells, TNF-α-induced matrix metalloproteinase-9 (MMP-9), an extracellular matrix–remodeling inflammatory mediator, was associated with increased H3K9 acetylation at the MMP9 promoter and enhanced NF-κB/AP-1 binding. Interestingly, treatment with 2-(1H-indol-3-ylcarbonyl)-4-thiazolecarboxylic acid methyl ester (ITE), an endogenous AhR ligand, reduced TNF-α-induced MMP-9 expression by decreasing promoter H3K9 acetylation and limiting NF-κB/AP-1 recruitment (60). As many microbiota-derived tryptophan metabolites also act as AhR ligands, this finding raises the possibility that microbial tryptophan metabolism may similarly influence inflammatory chromatin states in monocytic cells, although direct evidence for this metabolite-specific mechanism in macrophages remains to be established.

SCFAs can also imprint antimicrobial competence without provoking inflammatory pathology. During monocyte-to-macrophage differentiation, butyrate-driven HDAC3 inhibition reprograms metabolic and transcriptional circuits, including AMPK/mTOR signaling, reactive oxygen species (ROS) production, and antimicrobial effectors such as S100A8/S100A9, without overt inflammatory activation (61). In parallel, acetate suppresses cytokine storm via an ACSS2-dependent epigenetic–metabolic axis that increases histone acetylation, inhibits HIF-1α-dependent glycolysis, and reduces acetylation of NF-κB p65, demonstrating that SCFAs can restrain systemic immunopathology through pathways other than GPCR signaling (62).

Meanwhile, SCFA-mediated chromatin regulation can also dampen antiviral transcriptional outputs rather than uniformly enhancing host defense. Butyrate has been reported to repress type I interferon responses via HDAC inhibition and to suppress a substantial fraction of interferon-stimulated genes in macrophages, thereby increasing susceptibility to viral infection (63).

Collectively, these findings illustrate how locally derived metabolites orchestrate epigenetic modifications that optimize macrophage functions in a tissue- and context-specific manner and highlight that a broad repertoire of metabolites beyond butyrate can direct macrophage specialization.

#### 2.2.2 Dendritic cells

Dendritic cells (DCs) are indispensable for innate immune defense and for coordinating adaptive immunity, yet DCs are also implicated in the initiation and progression of diverse diseases. Continuously generated from the bone marrow, DCs diversify into multiple subsets that populate a wide range of tissues, including the skin, blood, lung, liver, gastrointestinal tract, and kidney, where these cells acquire distinct phenotypes and functions (64). To adapt to these diverse tissue niches, DC identity and responsiveness are shaped by epigenetic regulation (65). Within this framework, microbiota-derived metabolites have emerged as key local signals that drive epigenetic reprogramming and thereby modulate the developmental and functional diversity of DCs (66).

A major theme is that microbial metabolites impair DC development and induce an immature phenotype, ultimately driving the acquisition of a tolerogenic state. For example, butyrate and propionate were shown to impair DC development at the progenitor stage by entering cells via the transporter SLC5A8 and inhibiting HDAC1 and HDAC3, accompanied by altered activity of lineage-defining transcription factors, including PU.1 and RelB (67).

Consistent with this differentiation-level regulation, microbiota-derived metabolites appear to preferentially act on intestinal conventional DC2 (cDC2), whose substantial plasticity allows them to readily respond to environmental cues and adopt context-specific transcriptional programs (68). Thus, butyrate selectively restricts their lineage development, proliferation, and antigen-presenting capacity through an HDAC3-dependent mechanism independent of canonical SCFA-sensing GPCRs, thereby promoting a tolerogenic cDC phenotype that supports intestinal homeostasis (69).

Similarly, in human monocyte-derived DCs, butyrate induces an immature phenotype, characterized by impaired differentiation, reduced T cell stimulatory capacity, and enhanced endocytosis, thereby limiting DC maturation. Meanwhile, butyrate also promotes a cytokine shift toward a tolerogenic state, characterized by increased IL-10 production and reduced IL-12 and IFN-γ. Although these studies support a role for butyrate in promoting immature and tolerogenic DC states, the precise causal chromatin features were not fully mapped in these reports (70, 71).

More recent work further demonstrated that butyrate suppresses LPS-induced maturation and metabolic reprogramming in human monocyte-derived DCs and promotes a tolerogenic phenotype by inducing RALDH1. This effect depends on both HDAC inhibition and GPR109A signaling, which together enhance the retinoic acid-producing capacity of DCs. In parallel, butyrate-mediated HDAC inhibition reduces CD1 expression and suppresses LPS-induced IL-12 and IL-6 production, thereby limiting Th1-polarizing capacity. Together, these changes enable butyrate-conditioned DCs to restrain Th1 polarization and instead drive naïve CD4^+^ T cells toward IL-10-producing type 1 regulatory T cells (Tr1) (72, 73).

In addition to HDAC regulation, non-SCFA microbial metabolites also instruct DC tolerance through methylation-linked pathways: gut bacterial L-lysine activates the serine, glycine, one-carbon (SGOC) metabolic module to increase SAM availability and drive disruptor of telomeric silencing 1-like (DOT1L)-mediated H3K79me2 deposition at tolerogenic gene promoters, including *Tgfb* and *Stat3*, thereby restoring immune homeostasis across inflammatory disease models (74).

Conversely, epigenetic remodeling can also potentiate selected immunostimulatory functions in a context-dependent manner. SCFAs promote dendrite elongation through HDAC inhibition and activation of SFK/PI3K/Rho-family GTPase-dependent actin polymerization, thereby enhancing antigen uptake and presentation independently of canonical SCFA-sensing GPCRs (75). Similarly, *Lactobacillus plantarum*-derived indole-3-lactic acid (ILA) increases IL12a expression in DCs by enhancing H3K27ac at regulatory regions, thereby priming CD8+ T cell immunity and restraining colorectal tumorigenesis, exemplifying how aromatic microbial metabolites can drive locus-specific histone acetylation to amplify DC-driven antitumor responses (76).

Collectively, these findings position DCs as dynamic interpreters of tissue metabolic cues, whose epigenetic plasticity enables fine-tuned immune calibration, thereby balancing tolerance and stimulation in response to commensal or pathogenic contexts (66, 77).

#### 2.2.3 T cells and B cells

In contrast to rapidly responding innate immune cells, adaptive lymphocytes such as T and B cells acquire long-lived epigenetic programs that support effector and memory differentiation in response to microbiota-derived metabolites. Rather than acting solely as acute immunomodulators, these metabolites couple metabolic inputs to chromatin and transcriptional circuits that govern cytotoxic function, stemness, recall capacity, and regulatory differentiation.

In glucose-restricted CD8^+^ T cells, acetate rescues effector activity by fueling ACSS2-dependent acetyl-CoA production, thereby increasing histone acetylation, chromatin accessibility, and elevating IFN-γ expression (17). Butyrate likewise reinforces cytotoxic T lymphocyte (CTL) programs through GPCR-independent inhibition of HDACs, increasing histone H4 acetylation at CTL-associated promoters and augmenting IFN-γ and granzyme B production (78).

In addition to effector function, metabolite-driven programs can reinforce stemness and durability. Lactate promotes stem-like CD8^+^ T cell programs via an acetylation-linked axis at the Tcf7 regulatory landscape, whereas butyrate enhances memory potential and recall capacity of antigen-activated CD8^+^ T cells (79, 80). Consistent with this concept, the microbiota-derived tryptophan metabolite indole-3-propionic acid (IPA) has been shown to enhance immunotherapy efficacy by promoting T cell stemness across cancer contexts, further supporting the idea that microbial metabolites can shape durable T cell states (81).

Conversely, microbiota-derived metabolites impose epigenetic programs that bias T cell differentiation toward regulatory states and dampen inflammatory outputs in a context-dependent manner. SCFAs produced by commensal *Clostridia* act as HDAC inhibitors to promote Foxp3^+^ regulatory T cell (Treg) differentiation and IL-10 expression, whereas folate produced by commensals such as *Bifidobacterium* and *Lactobacillus* intersects with methylation-dependent pathways that support Treg survival and the Th17/Treg balance, including miRNA-linked regulation (82).

In addition to canonical SCFAs, pentanoate reprograms lymphocytes through dual epigenetic mechanisms: HDAC inhibition, which suppresses IL-17A, and enhanced oxidative metabolism, which increases acetyl-CoA availability to support HAT activity and IL-10 induction, illustrating how microbial metabolites can simultaneously constrain inflammatory cytokines and promote regulatory cytokines (83). However, metabolite-sensitive epigenetic regulation is not uniformly homeostatic and can become maladaptive under conditions of metabolic dysregulation. In T cells, impaired activity of succinate dehydrogenase (SDH), the mitochondrial complex II enzyme that converts succinate to fumarate, causes succinate to accumulate relative to α-ketoglutarate, thereby altering chromatin accessibility and transcriptional signatures and promoting PRDM1-associated Th1 and Th17 inflammatory differentiation. This finding highlights how disrupted intracellular metabolite balance can shift T cell programs toward inflammation rather than regulation (84).

Also, in CD4+ T cells, disruption of polyamine synthesis or hypusine formation rewires TCA cycle metabolism, leading to altered histone acetylation, broader chromatin dysregulation, and impaired helper T cell lineage fidelity (21).

Together, these findings broaden the concept of metabolite-sensitive chromatin regulation beyond SCFAs, but whether microbiota-derived succinate or polyamines make equivalent contributions to these T cell epigenetic programs remains unclear.

Compared with T cells, fewer studies have examined how microbiota-derived metabolites epigenetically regulate B cell biology. Nonetheless, available evidence indicates direct control of humoral immunity, particularly antibody diversification and plasma cell differentiation. Dietary fiber-derived SCFAs, particularly butyrate and propionate, epigenetically reprogram B cells by inhibiting HDAC activity and increasing histone acetylation at miRNA host genes, leading to induction of miRNAs that target AID and Blimp-1. This B cell-intrinsic pathway restrains antibody responses, including autoantibody production, by limiting class-switch recombination, somatic hypermutation, and plasma cell differentiation (50, 85).

Collectively, these studies indicate that microbiota-derived metabolites durably tune adaptive immunity by linking metabolic availability to epigenetic control of effector and regulatory gene circuits in T cells, while imposing parallel chromatin- and miRNA-linked constraints on B cell antibody diversification and plasma cell differentiation.

#### 2.2.4 Innate lymphoid cells

Innate lymphoid cells (ILCs) are predominantly tissue-resident immune populations that orchestrate local immunity by rapidly sensing and responding to environmental cues, including cytokines. Traditionally, ILCs were distinguished from T and B lymphocytes by the associated lack of antigen-specific receptors and were considered devoid of immunological memory (86, 87).

However, recent studies show that tissue-adapted ILC2s acquire memory-like properties following cytokine tuning driven by intestinal microbiota-derived metabolites such as succinate, thereby reinforcing local immune protection (88, 89). Mechanistically, this adaptation is accompanied by enhanced chromatin accessibility at effector- and memory-associated loci, underscoring microbial metabolite-driven epigenetic remodeling as a key determinant of the functional plasticity of tissue-resident ILCs (89).

Microbiota-derived metabolites also remodel subset-specific ILC functions by engaging epigenetic mechanisms that calibrate lineage-appropriate effector programs. Indeed, differentiation appears to be regulated by DNA hydroxymethylation in ILC1s and the associated precursors.

In this context, ten–eleven translocation 1 (TET1), an enzyme that converts DNA methylation into DNA hydroxymethylation, binds to the *Tgfbr1* promoter and promotes hydroxymethylation at this locus. During the postnatal period, microbiota-driven reduction of bile acids, particularly cholic acid, suppresses TET1 expression and DNA hydroxymethylation, promoting ILC1 expansion. In contrast, in adult mice, TET1 restrains ILC1 hyperactivation, thereby helping to maintain intestinal homeostasis and limit susceptibility to intestinal inflammation (90).

In ILC2s, which are specialized for type 2 inflammation, butyrate suppresses proliferation and IL-5 and IL-13 production through HDAC-inhibitory activity, providing a chromatin-based mechanism to restrain allergic responses (91). Consistent with a gut–lung axis, diet- and microbiota-derived butyrate further limits pulmonary ILC2 expansion and GATA3-dependent effector programs, accompanied by increased acetylation at the nuclear factor, interleukin 3 regulated (NFIL3) promoter, in a manner consistent with HDAC inhibition, linking SCFA-driven chromatin remodeling to reduced type 2 pathology (92).

In ILC3s, which are specialized for barrier defense, SCFAs enhance IL-22 production by coupling metabolite sensing to chromatin remodeling. This process requires both HDAC inhibition and GPR41 signaling to increase histone modification-associated chromatin accessibility at the *Il22* promoter and to potentiate HIF-1α-dependent transcription (93).

In addition to subset-intrinsic regulation, metabolite-driven epigenetic mechanisms may also regulate the balance between ILC lineages within tissues. A recent study showed that intestinal ILC2s express high levels of the AhR, supported by a gut-adapted chromatin architecture at the *Ahr* locus (94). Thus, AhR signaling sustains chromatin accessibility through a cell-intrinsic positive feedback loop. Functionally, AhR suppresses ILC2 effector programming by reducing expression of the IL-33 receptor ST2 (IL1RL1) and type 2 mediators such as IL-5, IL-13, and amphiregulin, while supporting the maintenance and antibacterial effector function of ILC3s, thereby positioning AhR as a chromatin-linked node that helps set the intestinal ILC2–ILC3 balance (94, 95).

Given that multiple gut microbiota-derived tryptophan metabolites act as AhR ligands, these findings raise the possibility that microbial ligand availability tunes the balance between ILC subsets through chromatin-level regulation.

### 2.3 Epigenetic imprinting by the microbiota: from trained innate immunity to adaptive memory

“Trained immunity” refers to a form of innate immune memory in which prior stimulation induces persistent epigenetic and metabolic remodeling, allowing innate immune cells or their progenitors to respond differently to later challenges. This memory-like property challenges the classic view that immunological memory is exclusive to adaptive lymphocytes and has been described across myeloid cells, natural killer (NK) cells, and hematopoietic stem/progenitor compartments (96).

As evidence accumulates that microbiota-derived cues durably shaped epigenetic set points, trained immunity has emerged as a useful framework for understanding how microbial signals tune baseline inflammatory tone and host defense readiness (96).

On the proinflammatory side, microbial ligands induce canonical trained immunity, characterized by heightened secondary cytokine responses and stable epigenetic changes. Among these, fungal β-glucan is a prototypical inducer of training, alongside LPS. β-glucan increases H3K4me3 at the promoters of innate immune genes through a Dectin-1/Raf-1 signaling axis (97). This trained state is further sustained by metabolic rewiring, including glycolysis, glutaminolysis, and cholesterol synthesis. In particular, glutaminolysis-derived fumarate accumulates and inhibits KDM5 histone demethylases, thereby reinforcing permissive H3K4 methylation landscapes required for trained responses (98).

In chronic disease settings, the microbiota-derived uremic toxin indoxyl sulfate drives AhR-dependent immune-metabolic and epigenetic rewiring in monocytes and macrophages, amplifying proinflammatory cytokine production and positioning trained immunity as a pathogenic amplifier in conditions such as chronic kidney disease (99). Importantly, trained immunity is not uniformly inflammatory: accumulating evidence supports the existence of anti-inflammatory or homeostatic “innate memory-like” states shaped by commensals and the associated metabolites, often discussed in the context of trained tolerance or homeostatic reprogramming (96). At the differentiation level, butyrate-driven HDAC3 inhibition imprints macrophage programs that enhance antimicrobial competence while restraining inflammatory activation, emphasizing a distinct mode of long-term programming compared with classical proinflammatory training (61).

In parallel, metabolite-driven epigenetic remodeling is increasingly recognized as a key determinant of adaptive immune memory, functionally linking microbial metabolism to durable lymphocyte fate decisions (100). For example, lactate provides an acylation-coupled metabolic axis that promotes CD8^+^ T cell stemness by inhibiting HDAC activity, enhancing H3K27 acetylation at the Tcf7 super-enhancer, and inducing TCF7/TCF-1 expression, thereby potentiating antitumor responses *in vivo*. This illustrates how local metabolite landscapes can imprint memory-like potential through chromatin remodeling (79).

Similarly, microbiota-derived butyrate strengthens the memory potential and recall capacity of antigen-experienced CD8^+^ T cells via GPR41/GPR43-mediated sensing, supporting a model in which commensal metabolites guide durable T cell fates even when the precise chromatin marks remain incompletely defined (80).

Collectively, local immunity relies on rapid, tissue-specific responses shaped by the niche. Accordingly, epigenetic imprinting that preconfigures responses to secondary challenge, manifesting as trained immunity in innate cells and durable adaptive memory in lymphocytes, is a central determinant of barrier protection and inflammatory tone (10, 96). Since microbiota-derived metabolites tune these chromatin set points by modulating epigenetic enzymes and metabolic cofactor availability, the epigenome–microbiota axis represents a rational target for steering local immune readiness and tissue homeostasis.

## 3 Impacts of the epigenome–microbiota axis in tissue-specific local immunity

**Figure 2:**
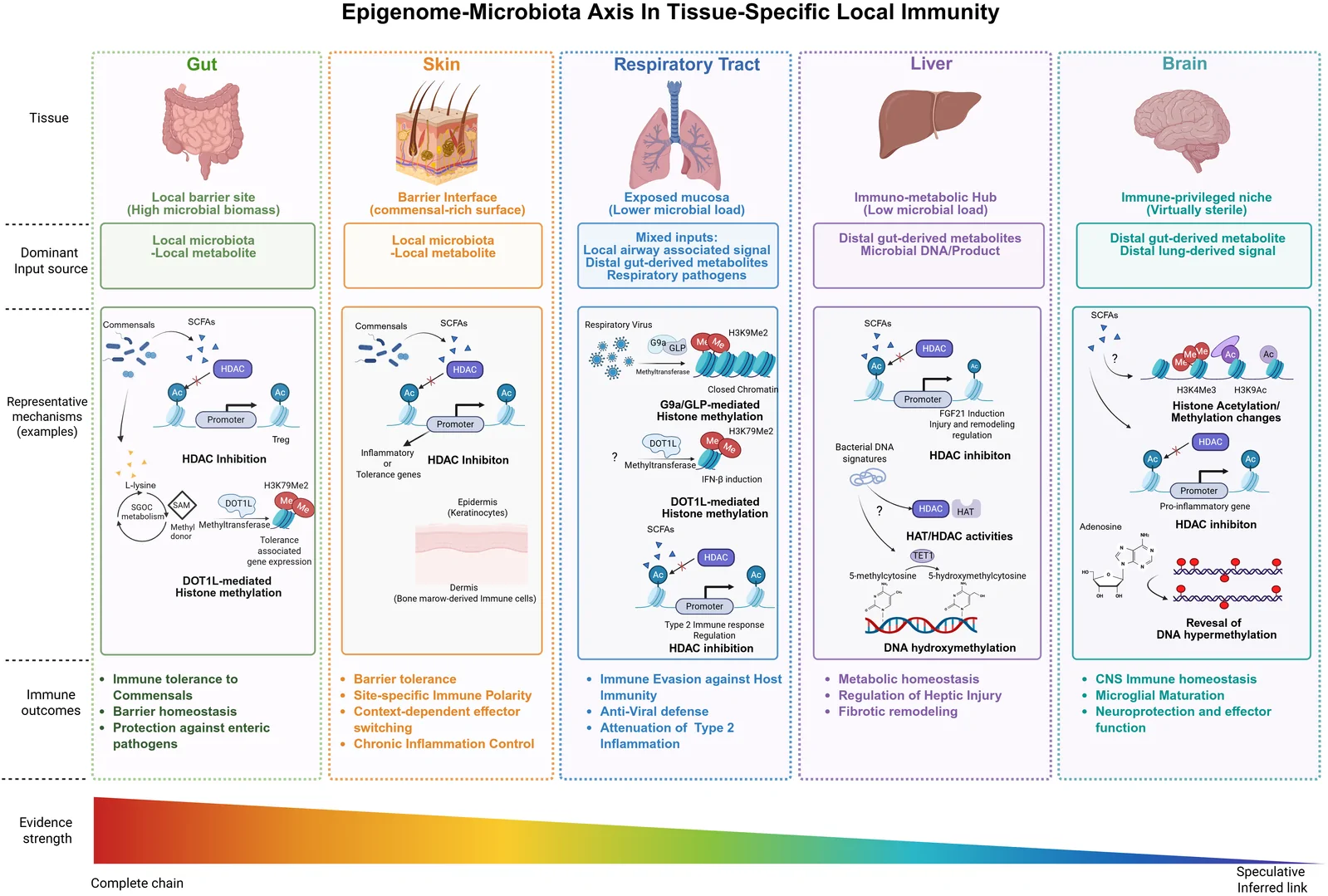
Epigenome-microbiota axis in tissue-specific local immunity. Schematic overview illustrating how local and distal microbiota-derived metabolites, microbial DNA/product, and pathogen-associated signals regulate tissue-specific immune programs through distinct epigenetic mechanisms. Each tissue panel summarizes the dominant microbial or metabolite input source, representative epigenetic mechanism, and associated immune outcome. In the gut, local microbiota-derived metabolites such as SCFAs and L-lysine support immune tolerance and barrier homeostasis through HDAC inhibition and DOT1L-mediated histone methylation. In the skin, local commensal-derived SCFAs regulate HDAC-dependent chromatin states in epidermal keratinocytes and deeper immune-cell compartments, contributing to barrier tolerance, site-specific immune polarity, and context-dependent inflammation. In the respiratory tract, mixed inputs, including local airway-associated signals, distal gut-derived metabolites, and respiratory pathogens, modulate antiviral defense, immune evasion, and type 2 inflammation through G9a/GLP-mediated histone methylation, DOT1L-mediated histone methylation, and HDAC inhibition. In the liver, distal gut-derived metabolites and microbial DNA/products are associated with HDAC-dependent injury and remodeling responses, altered HAT/HDAC activities, and DNA hydroxymethylation, contributing to metabolic homeostasis, hepatic injury regulation, and fibrotic remodeling. In the brain, distal gut- or lung-derived signals and microbial metabolites are linked to histone acetylation/methylation changes, HDAC inhibition, and reversal of pathological DNA hypermethylation, thereby influencing CNS immune homeostasis, microglial maturation, neuroprotection, and effector function. The evidence-strength gradient indicates the relative completeness of the causal chain, ranging from complete microbiota–metabolite–epigenome–immune axis to speculative or inferred inter-organ links. Abbreviations: CNS, central nervous system; DOT1L, disruptor of telomeric silencing 1-like; G9a/GLP, euchromatic histone lysine methyltransferase 2/1; HAT, histone acetyltransferase; HDAC, histone deacetylase; SCFA, short-chain fatty acid.

Next, to define how microbial metabolite-driven, tissue-specific functions are integrated across local immune cell populations to maintain each tissue niche. This perspective shifts the focus from epigenetic regulation in individual cells to tissue-level organization, highlighting how the microbiota–epigenome axis ultimately sustains local immunity as a coordinated niche program (**Figure 2**).

### 3.1 Gut: local microbiota-local metabolite effects

The gut represents the clearest example of local microbiota-local metabolite regulation, because dense commensal communities generate metabolites directly within the tissue niche in which immune and epithelial cells are continuously exposed. These metabolites provide local epigenetic inputs that calibrate immune set points, enabling tolerance toward resident microbes while preserving protective inflammatory responses against invasive pathogens (8, 101).

Given the central role of Foxp3^+^ Tregs in intestinal homeostasis, SCFAs are a prominent class of metabolites that promote tissue tolerance. SCFAs support the differentiation and function of peripheral colonic Tregs through HDAC inhibition, which increases histone acetylation at the *Foxp3* promoter and enhancer regions and enhances Foxp3 protein acetylation, thereby increasing protein stability and functional activity. These effects can be T cell-intrinsic and can also be reinforced by DC programs, including HDAC-sensitive repression of proinflammatory regulators such as RelB (102–104).

Beyond SCFAs, microbiota-modified secondary bile acids also support intestinal Treg programs through chromatin-linked mechanisms. The bacterial bile acid metabolite isoallolithocholic acid (isoalloLCA) promotes the differentiation of naïve CD4+ T cells into Tregs by enhancing NR4A1 binding and establishing a permissive chromatin state at the Foxp3 locus, thereby reinforcing the transcriptional program required for Treg induction. This example extends the gut tolerogenic metabolite axis beyond HDAC-inhibitory SCFAs and highlights secondary bile acids as additional microbiota-derived epigenetic cues that shape mucosal tolerance (105).

In addition to targeting Tregs, microbiota-derived metabolites also reset highly responsive innate immune compartments toward a restrained baseline. In intestinal macrophages, n-butyrate induces a hyporesponsive surveillance state through HDAC inhibition, limiting the induction of inflammatory mediators independently of canonical GPCR signaling (58). In addition to SCFAs, non-SCFA metabolites can also epigenetically program tolerogenic antigen-presenting cells (APCs). For example, gut commensal-derived L-lysine rewires SGOC metabolism and promotes DOT1L-linked H3K79me2 deposition at tolerogenic gene loci, including *Tgfb* and *Stat3*, thereby reinforcing mucosal homeostasis across inflammatory settings (74).

Collectively, microbiota-derived metabolites establish a tissue-adapted homeostatic baseline through epigenetic programming, enabling immune tolerance without compromising protective responsiveness.

### 3.2 Respiratory tract: local airway associated signals, distal gut derived metabolite effects, and pathogen induced epigenetic remodeling

The respiratory tract is a highly exposed mucosal surface that interfaces directly with the external environment and serves as a major entry site for airborne pathogens, including respiratory viruses and bacteria, as well as allergens, pollutants, and particulate matter (106). These features make respiratory immune regulation difficult to attribute to any single factor. Rather, respiratory epigenetic regulation likely reflects the convergence of multiple inputs. Local airway-associated microbial signals may shape epithelial and immune-cell states within the respiratory mucosa. In parallel, gut–lung axis studies indicate that metabolites generated by the dense commensal niche of the gut can also influence lung immunity through systemic or inter-organ routes (107). In addition, as noted above, infection-driven signals can independently induce chromatin remodeling and should be distinguished from commensal metabolite-driven programming.

This mixed input landscape helps explain why epigenetic regulation in the respiratory tract has been studied largely in the context of antiviral immunity. Importantly, the effects of chromatin remodeling are not uniformly protective and can be either proviral or antiviral depending on the virus, the cell type, and the timing of the response.

From the pathogen side, respiratory viruses actively rewire host chromatin to suppress interferon responses and enhance replication. Influenza virus NS2 antagonizes the antiviral chromatin regulator H1C to reduce IFN-β production, whereas SARS-CoV-2 employs NSP1 and ORF8 to impose repressive chromatin states through G9a/GLP-mediated H3K9 dimethylation and histone mimicry, respectively (108–110). Likewise, RSV enhances HDAC2-associated histone deacetylation in bronchial epithelial cells, a mechanism that may contribute to variation in disease severity, while HDAC inhibition restores antiviral signaling and reduces both viral replication and airway inflammation (111).

Conversely, to counteract virus-driven epigenetic immune evasion, host cells deploy intrinsic epigenetic mechanisms that sustain antiviral defense. Here, DOT1L-mediated H3K79 methylation supports innate antiviral immunity by promoting RIG-I-dependent IFN-β induction, and this protective role is underscored by the finding that DOT1L inhibition disrupts this pathway and enhances influenza virus replication (112). However, while these studies emphasize direct host–virus epigenetic interactions, none fully capture the local immune context of infection, particularly given that respiratory viral infections also perturb the lung microbiota and may reshape tissue-local immunity (106, 113). Mechanistic connections to host epigenetic regulation remain comparatively underdeveloped, in part because the lung microbiome is a low-biomass ecosystem that was long presumed sterile and is technically challenging to profile without contamination-aware approaches (114).

Despite these constraints, emerging evidence suggests that microbial metabolites can influence local respiratory immunity through epigenetic mechanisms. For instance, butyrate, an HDAC inhibitor produced by microbial fermentation, promotes influenza replication in lung epithelial cells by repressing a subset of antiviral ISGs (63), yet can also alleviate airway type 2 inflammation by constraining pulmonary ILC2 expansion and GATA3-dependent effector programs, reducing IL-5 and IL-13 in asthma/Chronic obstructive pulmonary disease (COPD)-relevant models (91, 92).

Collectively, these findings indicate that epigenetic regulation in the respiratory tract arises from intersecting local airway-associated signals, distal gut-derived metabolites, and pathogen-induced chromatin remodeling. Compared with the gut, however, direct evidence for a complete local microbiota-local metabolite-epigenome axis in local immunity remains limited, making this an important frontier for future respiratory epigenetic research.

### 3.3 Skin: local microbiota-local metabolite effects

The skin provides another barrier-site example in which local microbial communities can generate metabolites or microbial products that act directly on keratinocytes and tissue-resident immune cells. Unlike internal mucosal sites, the skin is a stratified, relatively closed epithelial surface with limited exposure to systemic metabolite flux and a comparatively low contribution from distal microbial niches. Instead, it is continuously colonized by stable, site-specific commensal communities that reside in close physical proximity to keratinocytes. In this context, keratinocytes function not only as a structural barrier but also as a primary immune-sensing interface that must maintain tolerance to resident microbes while remaining poised to respond to environmental threats. These features make local microbiota-derived signals particularly relevant for cutaneous immune regulation, positioning locally produced metabolites as context-dependent epigenetic cues that calibrate keratinocyte activation states and downstream immune response.

In epidermal keratinocytes, *Cutibacterium acnes* (*C. acnes*)*-*derived SCFAs, particularly propionate and valerate, are generated under lipid-rich, hypoxic conditions and inhibit HDAC activity. In sebaceous low-oxygen follicular niches, this effect breaks epidermal tolerance to TLR2 and TLR3 ligands through an HDAC8/9-dependent pathway, increasing histone acetylation and inflammatory gene expression (115, 116). Notably, the same SCFA–HDAC axis shows site-specific immunologic polarity, promoting inflammatory programs in epidermal keratinocytes while suppressing inflammatory outputs in deeper bone marrow-derived immune cells, thereby illustrating how commensal metabolism differentially tunes immune set points across skin niches (115, 116). Together, these observations raise the possibility that follicular occlusion and sebum accumulation may enhance SCFA production by *C*. *acnes*, shifting keratinocyte chromatin toward a hyperacetylated, proinflammatory state. Such epigenetic reprogramming could precipitate cutaneous inflammation and disrupt tolerance to resident commensals.

Not all skin microbiota-associated epigenetic effects, however, have been linked to a defined metabolite. The skin also harbors commensal-specific CD8^+^ T cells that are epigenetically primed for rapid effector switching. Commensal-specific Tc17 cells retain a type 17 antimicrobial program while also maintaining a poised type 2 state, characterized by GATA3-associated chromatin accessibility at type 2 loci such as *Il5* and *Il13* and by preformed type 2 cytokine mRNA, thereby enabling rapid transition to type 2 responses after tissue injury (115). Whether commensal metabolites directly shape this poised chromatin landscape remains an important open question (117).

Conversely, in atopic dermatitis (AD)-like settings, often driven by the alarmin IL-33, commensal- and sebum-associated SCFAs can act primarily as anti-inflammatory cues in keratinocytes. Topical propionate attenuates AD-like inflammation by suppressing keratinocyte IL-33 production through HDAC3 inhibition, with involvement of AhR signaling (118). Similarly, butyrate attenuates *S. aureus*-aggravated skin inflammation by suppressing keratinocyte-derived IL-33 through HDAC3 inhibition, as demonstrated in human keratinocyte cells and an AD-like mouse model (119).

Thus, SCFA-sensitive chromatin states appear to function as tunable regulators of cutaneous immune tone, with directionality determined by niche, context, and delivery route.

### 3.4 Liver: distal gut-derived metabolite effects and microbial DNA/product associations

In contrast to the gut and skin, the liver should be interpreted primarily within a gut–liver axis framework rather than as a high-biomass local microbiota niche. Nevertheless, the liver is highly relevant to local immunity because it is continuously exposed to gut-derived metabolites and microbial products through the portal circulation, allowing extrahepatic microbial signals to shape tissue-local immune and metabolic programs within the hepatic microenvironment. Accordingly, hepatic immune and metabolic regulation can be influenced by at least two microbiota-associated input routes: distal gut-derived metabolites, such as circulating SCFAs, and microbial DNA or microbial products that translocate from the intestinal compartment. These routes should be distinguished from direct local microbiota–local metabolite programming.

Among distal gut-derived metabolites, butyrate provides the clearest example of chromatin-associated hepatic regulation, as it can reprogram hepatic metabolic gene networks through HDAC3-dependent mechanisms. In hepatocytes, sodium butyrate induces FGF21 via HDAC3 inhibition, thereby promoting ketogenesis and fatty acid oxidation (120). Fecal microbiota transfer (FMT)-associated increases in circulating butyrate have been linked to reduced HDAC3 activity in liver sinusoidal endothelial cells (121).

However, microbiota–epigenome coupling in the liver does not appear to operate uniformly at a global level. Microbiota depletion can markedly reduce luminal and portal SCFAs without broadly altering global hepatic histone acetylation, suggesting that microbiota-dependent epigenetic regulation in the liver is more likely to be locus- and context-specific than globally distributed (122).

Butyrate-mediated HDAC regulation may also influence hepatic injury and fibrotic remodeling.

In hepatic ischemia-reperfusion injury, butyrate preserves histone acetylation and limits inflammatory damage, including Kupffer cell NF-κB activation (123, 124). Hepatic stellate cells (HSCs), quiescent vitamin A-storing pericytes that become activated after injury, also appear to be epigenetically targetable, as HDAC inhibition suppresses myofibroblastic differentiation, proliferation, and fibrosis-related gene expression (125, 126).

A second line of evidence involves microbial DNA/product associations rather than defined metabolite-mediated mechanisms. In patients with metabolic dysfunction-associated steatotic liver disease (MASLD), hepatic HAT and HDAC activities, as well as global 5-hydroxymethylcytosine (5-hmC) levels, are associated with liver bacterial DNA signatures. Mediation analysis further suggests that selected taxa may contribute to steatosis severity, in part, through altered HAT activity (127). These findings link microbiota-associated signatures to hepatic epigenetic state, but they do not establish a complete local liver microbiota-metabolite-epigenome axis.

Together, as discussed above, current evidence suggests that liver-associated microbiota–epigenome interactions are best understood as gut–liver axis effects involving circulating metabolites and microbial DNA/product-associated signals, rather than as direct proof of local microbiota-dependent epigenetic programming within a resident liver microbial niche. Defining which microbial metabolites or microbial products reach specific hepatic cell populations, and whether they establish durable, locus-specific chromatin programs, will be essential for translating microbiota–epigenome interactions into therapeutic strategies.

### 3.5 Brain: distal gut-brain axis and lung-brain axis effects

The brain has long been regarded as an immune-privileged organ; however, this view has been revised following the discovery of functional meningeal lymphatics and the recognition that diverse peripheral immune cells reside in and actively interact within the meninges (128, 129). Therefore, the central nervous system (CNS) is now better conceptualized as an immune-specialized niche in which inflammatory responses are not absent, but instead tightly controlled, because excessive immune signaling can directly disrupt neural circuits, synaptic integrity, and cell survival (128).

A central cellular mediator of this regulated immune niche is the microglial population, the dominant tissue-resident immune cell type in the CNS (130). Although historically described as “resting,” microglia are now understood to be highly dynamic cells that continuously survey the parenchyma and contribute to circuit refinement and host defense in response to local perturbations, as demonstrated by *in vivo* imaging and functional studies (130).

However, the brain is not currently considered an established resident microbiota niche; therefore, microbiota-associated epigenetic regulation in the CNS should be interpreted primarily within a microbiota–gut–brain axis framework. This framework includes distal microbial metabolites, circulating microbial products, peripheral immune mediators, and neural or endocrine signaling that can influence CNS-resident immune cells such as microglia (131, 132). In parallel, conceptual frameworks such as the gut–brain axis and, more recently, the lung–brain axis highlight that CNS immune tone can be shaped by distal mucosal ecosystems, motivating growing interest in how microbiota-derived metabolites influence microglial states (133, 134). Consistent with this view, germ-free conditions are associated with an immature microglial phenotype, metabolic defects, and stable changes in histone modification landscapes, including H3K4me3 and H3K9ac at loci linked to metabolic gene programs (135). In the same study, microbiota-derived acetate emerged as a key SCFA that can modulate these programs. Moreover, in an Alzheimer’s disease mouse model, acetate was linked to altered microglial amyloid-β phagocytosis and disease progression. However, a complete causal chain from acetate availability to specific histone changes remain to be defined (135).

Similarly, sodium butyrate attenuates microglia-driven neuroinflammation after ischemic stroke via HDAC inhibition–associated chromatin regulation, decreasing proinflammatory gene programs while enhancing anti-inflammatory pathways through an IL-10/STAT3 axis and associated changes in H3K9ac (136).

Collectively, microbiota-derived SCFAs have been reported to influence CNS immune tone, and low levels of selected SCFAs have been detected in cerebrospinal fluid (137). Although blood–brain barrier transport has been proposed to involve monocarboxylate transport systems, the extent, route, and physiological relevance of SCFA access to the CNS remain context-dependent and require further validation (138). Therefore, manipulating peripheral SCFA availability may represent a plausible, but still incompletely defined, strategy to recalibrate microglial epigenetic set points in settings of maladaptive neuroinflammation.

Although most microbiota-focused studies in CNS have centered on microglial maturation and inflammatory tone, metabolite-driven epigenetic regulation in the brain is not limited to microglia. Purine metabolism provides one example of how metabolite availability can reshape neural epigenetic states.

Adenosine augmentation therapy was shown to reverse pathological DNA hypermethylation in the epileptic brain and suppress structural remodeling associated with epileptogenesis. However, this study did not establish a microbiota-derived source of adenosine or define an immune cell-specific epigenetic mechanism and therefore should be interpreted as evidence that purine metabolites can regulate CNS epigenetic states rather than as a complete microbiota-metabolite-epigenome chain in local immunity (139). Together, current evidence suggests that brain-associated microbiota-epigenome interactions are best understood as distal gut-brain or lung-brain axis effects that modulate local neuroimmune states, rather than as direct resident brain microbiota-driven programming. Future studies are needed to define how specific microbial metabolites reach distinct CNS niches and reshape cell-type-specific immune and neural epigenetic programs.

### 3.6 Key findings from human studies

Human studies support three broader conclusions regarding the microbiota–metabolite–epigenome axis. First, human-derived experimental systems demonstrate that metabolite exposure can directly alter epigenetic regulation and cellular function, extending this concept beyond animal models. In ex vivo-generated human monocyte-derived dendritic cells, butyrate promotes tolerogenic programming, whereas *C. acnes*-derived SCFAs enhance inflammatory responsiveness in human keratinocyte and sebocyte models (70–73, 115, 116). Evidence for metabolite-sensitive epigenetic regulation is not limited to isolated cell systems, as butyrate-dependent HDAC3 regulation has also been associated with altered tuft-cell differentiation in human intestinal organoids (140). Second, human evidence indicates that tissue-resident non-immune cells are important direct targets of microbial metabolite-driven epigenetic regulation. The responses observed in keratinocytes, sebocytes, and intestinal tuft cells suggest that the microbiota–metabolite–epigenome axis can shape local immunity not only through immune-cell-intrinsic programming but also by reprogramming non-immune cell compartments that regulate tissue immune environments (115, 116, 118, 140). This interpretation is consistent with the definition of local immunity adopted in this review. Third, patient-based studies extend these experimental findings to human disease by linking reduced cutaneous propionate availability to atopic dermatitis severity, liver bacterial DNA signatures to hepatic HAT and HDAC activities and global 5-hmC levels in MASLD, and intestinal microbial composition to DNA methylation patterns in human colonic tissue (118, 127, 141). Collectively, these findings support the relevance of metabolite-sensitive and microbiota-associated epigenetic regulation in humans and suggest that its effects on local immunity are mediated through coordinated regulation of both immune and tissue-resident non-immune cells.

## 4 Mechanistic principles underlying the context-dependent microbiota-metabolite-epigenome axis

The studies discussed above indicate that microbiota-derived metabolites should not be interpreted as molecules with fixed immunological functions. Instead, their effects emerge from the interaction between a metabolite, the responding cell, and the tissue environment. Thus, the same metabolite may promote tolerance, reinforce immune memory, suppress inflammatory pathology, or impair host defense depending on the biological context in which it is sensed. This context-dependent directionality provides a conceptual framework for reconciling apparently divergent findings across tissues and immune-cell subsets (8, 10, 11).

A first principle is that tissue context determines the functional meaning of a metabolite-induced epigenetic change. Each tissue niche differs in microbial biomass, metabolite concentration, oxygen tension, nutrient availability, barrier exposure, and dominant immune pressure. In high microbial-load barrier tissues, chronic exposure to microbial metabolites may favor the establishment of restrained homeostatic programs that prevent excessive inflammation against commensals (10, 11). In contrast, in tissues or disease states requiring rapid inducible defense, the same chromatin-modifying input may become detrimental if it dampens transcriptional programs required for acute antimicrobial or antiviral responses (58, 63). Therefore, tissue context determines whether epigenetic restraint is interpreted as tolerance, resolution, immune suppression, or impaired host defense.

A second principle is that cell identity and the baseline chromatin landscape determine the target specificity of metabolite action. Microbial metabolites do not act on a chromatin landscape that is uniform across all immune cells. Instead, they are interpreted through lineage-specific transcription factor networks, enhancer accessibility, and pre-existing epigenetic states (53, 102, 103). Therefore, regulatory T cells, macrophages, dendritic cells, epithelial cells, innate lymphoid cells, and memory T cells may all sense the same metabolite, but the genomic loci available for remodeling differs substantially among these populations. Even within a given cell type, metabolite-induced changes in histone acetylation, histone methylation, histone crotonylation, or DNA methylation are not functionally equivalent across the genome. Their consequences depend on whether the affected loci encode inflammatory mediators, regulatory cytokines, antiviral genes, alarmins, metabolic regulators, or memory-associated transcription factors (17, 27, 28, 60). Thus, cell identity defines the range of immune programs that can respond, whereas the local chromatin state determines which specific gene loci are remodeled and translated into functional immune outcomes.

A third principle is that timing and duration of exposure shape whether metabolite-driven epigenetic remodeling supports adaptive tissue or immune regulation or instead promotes maladaptive inflammatory states. Persistent low-level exposure can contribute to tissue adaptation by stabilizing durable transcriptional states (10, 96). In contrast, acute exposure during infection, injury, or inflammatory activation may interfere with rapidly inducible response programs (63). Similarly, early-life or chronic exposure may establish long-term immune set points, whereas transient exposure during an ongoing immune response may alter effector differentiation, cytokine production, or recall potential (80, 96). Thus, metabolite effects should be evaluated not only by concentration, but also by when and for how long the metabolite is encountered.

A fourth principle is that receptor, transporter, and enzyme expression determine which signaling route predominates. Many microbial metabolites can act through multiple mechanisms, including GPCR signaling, transporter-dependent uptake, metabolic conversion into cofactors, or direct regulation of chromatin-modifying enzymes (8, 17, 67, 73). The dominant pathway depends on the expression of receptors, transporters, and epigenetic enzymes in each cell type. For example, a metabolite may function primarily as a receptor ligand in one cell, as an HDAC inhibitor in another, and as a carbon source for acetyl-CoA-dependent chromatin modification in a third (17, 67, 73). This explains why assigning a single mechanism to a metabolite class is often insufficient.

Together, these principles argue that microbiota-derived metabolites act as conditional epigenetic inputs rather than universal immune activators or suppressors. Their functional direction is determined by the convergence of tissue niche, cell identity, exposure timing, receptor and transporter usage, metabolic state, and chromatin architecture. This framework also clarifies why causal interpretation requires more than simply showing that a metabolite alters an immune phenotype. A stronger mechanistic chain should define the microbial source, metabolite availability, sensing or uptake pathway, chromatin-modifying enzyme or epigenetic mark, target gene locus, responding cell type, and validated immune outcomes.

## 5 Unresolved mechanistic and translational questions

Although microbiota-derived metabolites are increasingly recognized as epigenetic regulators of local immunity, the field still faces several unresolved mechanistic and translational challenges. These include the disproportionate focus on SCFAs, incomplete causal evidence, limited human validation, and uncertainty regarding the safety of metabolite-based interventions.

### 5.1 Unresolved mechanistic questions

To date, microbiota-metabolite-epigenome research has focused predominantly on SCFAs, especially butyrate. This strong emphasis likely reflects both biological and technical factors. SCFAs are abundant microbial fermentation products, and their production is strongly linked to well-characterized commensal metabolic pathways, providing a relatively clear microbiota-driven source compared with many other metabolite classes. This defined microbial origin has made SCFAs particularly attractive for studies aiming to connect microbial activity with host epigenetic and immune outcomes. SCFAs can also be readily quantified in intestinal and systemic compartments, and can be experimentally manipulated through diet, antibiotic treatment, germ-free models, or direct metabolite supplementation (8, 104, 142). Most importantly, SCFAs produce clear and reproducible immune phenotypes across multiple immune-cell populations spanning innate and adaptive immunity (58, 85, 104). These features have made SCFAs particularly well suited for connecting the microbiota–metabolite–epigenome axis to defined immune phenotypes.

However, one important unresolved question is whether HDAC inhibition alone is sufficient to explain the broad immunological effects of SCFAs. Although butyrate and propionate are frequently interpreted as HDAC inhibitors, SCFAs can also act through GPCR signaling, transporter-dependent uptake, metabolic conversion into acetyl-CoA, altered HAT activity, mitochondrial metabolism, and non-canonical acylation pathways such as histone crotonylation. Therefore, SCFA-driven immune regulation should not be reduced to a single HDAC-dependent mechanism.

At the same time, SCFAs are not the only microbiota-derived metabolites that can shape immune epigenomes. This broader view requires greater attention to non-SCFA metabolites, including tryptophan derivatives, secondary bile acids, succinate, microbial vitamins, and one-carbon metabolism-related metabolites, to the microbiota–metabolite–epigenome–immune axis.

Despite this broader perspective, several limitations constrain current understanding. Only a few studies have delineated a complete causal axis linking a defined commensal taxon or consortium, a specific metabolite, and a corresponding chromatin modification to a validated immune outcome. Moreover, while correlations between metabolite exposure and epigenetic remodeling are increasingly reported, the molecular mechanisms that translate metabolite availability into locus- and cell-type-specific chromatin changes remain incompletely defined.

To clarify these differences, we classified representative studies according to the completeness of the microbiota–metabolite–epigenome causal chain, defined here by whether a study connects microbial source, metabolite availability, epigenetic mechanism or mark, target cell or locus, and validated immune outcome in local immunity (**Table 2**).

**Table 2:** Evidence for microbiota-metabolite-epigenome axis in local immunity.

Category name	Integrated criterion	Example chain	Unresolved step	Ref.
Highly integrated mechanistic evidence with an unresolved upstream step	Most components of the causal chain are experimentally connected, including the microbial source or commensal-derived metabolite, target locus, responding cell type, epigenetic modification, and immune outcome, but the upstream sensing, uptake, or intracellular metabolic pathway remains incompletely defined.	Mouse/Colon Tumors; DCs *L. plantarum*-derived ILA → increased H3K27ac at Il12a regulatory regions in DCs → increased IL-12A and enhanced CD8^+^ T cell-mediated antitumor immunity	Sensing, uptake, or intracellular metabolic pathway	(76)
		Mouse/Tumor CD8+ T cells *L. johnsonii* and *C. sporogenes*-derived IPA → increased H3K27ac at the Tcf7 super-enhancer region in CD8+ T cells → CD8+ Tpex/stemness program and enhanced anti-PD-1 antitumor immunity		(81)
		Mouse/Colon Tregs Commensal-derived butyrate → increased histone H3 acetylation at Foxp3 promoter/enhancer regions → Foxp3^+^ colonic Treg differentiation and intestinal immune tolerance	Sensing, uptake, or intracellular metabolic pathway and Microbial producer	(102)
		Mouse/Intestinal DCs Gut bacterial L-lysine → SGOC metabolism/SAM availability → DOT1L-dependent H3K79me2 at Tgfb and Stat3 loci → Tolerogenic DC programming and restored immune homeostasis		(74)
Partial mechanistic evidence with incomplete microbial or locus-level linkage	Metabolite-to-epigenetic mechanism-to-immune outcome is demonstrated, but microbial source, precise target locus, or locus-level chromatin validation remains incomplete.	Mouse/CD8+ T cells Acetate → ACSS2-dependent acetyl-CoA production → increased histone acetylation and chromatin accessibility → restored IFN-γ production and CD8^+^ T cell effector function under glucose restriction	Microbial source; Precise target locus or chromatin target	(17)
		Mouse/DCs Butyrate/propionate → SLC5A8-dependent uptake → HDAC1/HDAC3 inhibition with altered PU.1/RelB activity → impaired DC development and tolerogenic DC bias		(67)
		Human/Monocyte-derived DCs Butyrate → HDAC inhibition + GPR109A signaling → RALDH1 induction in human DCs → Tr1 priming		(73)
		Mouse/Macrophages Butyrate → HDAC3 inhibition and metabolic rewiring → S100A8/S100A9-associated antimicrobial program → enhanced macrophage bacterial killing without overt inflammatory activation		(83)
		Mouse/B cells Butyrate/propionate → HDAC inhibition at miRNA host genes → increased miRNAs targeting AID/Blimp-1 → reduced class-switch recombination, somatic hypermutation, plasma cell differentiation, and antibody responses	Microbial source; Sensing, uptake, or intracellular metabolic pathway	(85)
		Mouse/Intestine; ILC1s Microbiota-regulated cholic acid availability → TET1 expression/DNA hydroxymethylation at the Tgfbr1 locus → Altered intestinal ILC1 expansion and homeostasis		(135)
		Mouse/Macrophages Pentanoate → HDAC inhibition and increased acetyl-CoA/HAT activity → decreased IL-17A and increased IL-10 → regulatory lymphocyte programming	Microbial source; Target locus and Sensing, uptake, or intracellular metabolic pathway	(61)
Epigenetic remodeling observed, but causal metabolite link unresolved	Microbiota-associated epigenetic remodeling and immune changes are observed, but the specific causal metabolite, metabolite-dependent pathway, or direct metabolite-to-epigenetic mark remains incompletely defined.	Mouse/CNS; Microglia Germ-free or microbiota-altered state → Altered acetate -associated microglial metabolic fitness → changes in H3K4me3, H3K9ac at metabolic genes → altered microglial function	Causal metabolite; Sensing, uptake, or intracellular pathway, target locus, responding cell	(90)
		Human/Liver Liver bacterial DNA signatures in MASLD → Altered hepatic HAT and HDAC activity and 5-hmC levels → association with steatosis severity		(127)
		Mouse/Skin; CD8+ T cells Skin commensal-specific CD8^+^ T cells → poised chromatin accessibility at type 2 loci such as IL5 and IL13 → rapid type 2 switching after tissue injury	Causal metabolite; Sensing, uptake, or intracellular pathway, Epigenetic mechanism	(117)
		Mouse/Lung; Alveolar macrophages Segmented filamentous bacteria → Epigenetic reprogramming of alveolar macrophages → Enhanced phagocytosis and protection from post-influenza bacterial pneumonia		(153)
Speculative or inferred link	A microbiota/metabolite–epigenome–immune axis is proposed based on known biology or associative evidence, or parallel findings, but direct evidence connecting metabolite availability, epigenetic modification, and immune outcome is lacking.	Mouse/Intestine; ILCs Gut microbiota-derived tryptophan metabolites → possible AhR ligand availability → AhR-linked chromatin accessibility in ILCs → potential tuning of ILC2–ILC3 balance	Direct metabolite-to-chromatin causality	(94, 95)
		Mouse/CD8+ T cells Microbiota-derived butyrate → GPR41/GPR43-mediated effects on CD8^+^ T cell memory potential and recall capacity → possible epigenetic imprinting		(80)
		Inferred/CNS; Microglia Gut–brain or lung–brain microbial/metabolite axis → possible modulation of CNS immune tone → potential microglial epigenetic set-point regulation	Defined metabolite-to-epigenome causal chain	(133, 134)

Moreover, a related challenge is determining whether observed epigenetic changes are causal drivers of immune outcomes or secondary consequences of altered cell states, such as activation, differentiation, exhaustion, metabolic remodeling, or tissue adaptation.

Additionally, much of the mechanistic evidence derives from animal models, whereas human studies remain limited. Although germ-free mice, antibiotic-treated mice, fecal microbiota transfer models, and direct metabolite supplementation have been essential for defining mechanisms, they do not fully reproduce the complexity of human microbiota composition, diet, genetics, medication exposure, disease heterogeneity, or tissue accessibility (28, 143, 144).

Thus, translating mouse findings to human immunity will require longitudinal human cohorts, tissue-relevant sampling, cell-type-resolved epigenomics, and functional validation using human primary cells or organoid/co-culture systems.

### 5.2 Therapeutic opportunities and clinical considerations

A rapidly expanding body of disease-focused literature suggests that metabolite-driven epigenetic remodeling is not merely descriptive but represents a potential therapeutic axis. Moreover, gut-centered disorders such as inflammatory bowel diseases (IBDs), including ulcerative colitis (UC), share mechanisms implicated in asthma/COPD, AD, and neurodegenerative diseases, in which microbial metabolites can either reinforce protective tissue programs or induce maladaptive chromatin states.

These insights support the notion that metabolite–epigenetic interfaces could be targeted to fine-tune tissue-specific local immunity. Therapeutically, this framework suggests several complementary intervention strategies. Prebiotics may selectively enrich metabolite-producing commensals and increase the endogenous production of beneficial microbial metabolites, as demonstrated by the ability of inulin supplementation to promote colonic short-chain fatty acid production in humans (145). Alternatively, postbiotics or defined microbial metabolites could be administered directly to engage host receptors or chromatin-regulatory pathways; butyrate-mediated histone deacetylase inhibition and histone acetylation-dependent induction of colonic regulatory T cells provide a representative mechanistic example (102). Engineered probiotics offer a more programmable approach by producing metabolites or metabolic enzymes directly within a selected tissue niche, as illustrated by an engineered *Escherichia coli Nissle* strain that increased intratumoral L-arginine and enhanced antitumor T-cell immunity (146). FMT may restore broader microbial community functions rather than a single metabolite pathway, including the recovery of short-chain fatty acid and secondary bile acid metabolism observed in recurrent Clostridioides difficile infection (147). In parallel, tissue-targeted formulations, including metabolite-loaded or metabolite-derived nanoparticles, may improve local bioavailability while limiting systemic exposure and off-target effects (148).

Despite this therapeutic potential, such interventions require caution: because microbiota and metabolite landscapes are dynamic, context-dependent, and highly individualized, shaped by interindividual differences in microbial composition, diet, drug exposure, and genetics are therefore likely to produce substantial variation in tissue metabolite exposure and epigenetic responsiveness (2).

Accordingly, metabolite supplementation could have harmful effects if applied without accounting for tissue niche, disease stage, timing, dose, infection status, baseline metabolite availability, and patient-specific microbiota and epigenetic responsiveness. For example, a metabolite that supports tolerance or inflammatory resolution in one tissue may impair protective host defense or promote maladaptive immune programming in another context (149–151).

Therefore, the next phase of the field will require integrative, personalized frameworks that combine longitudinal metabolomics, cell-type-resolved epigenomics, and causal microbial genetics. Such strategies should enable metabolite-based interventions to be tailored to the host–microbiota state rather than implemented as uniform supplementation approaches.

## 6 Conclusions and future directions

Recent studies have shown that microbiota-derived signals can shape immune cell differentiation and function across diverse local tissue environments, either through direct host–microbe interactions at colonized surfaces or through systemic exposure to microbial metabolites and products in distal tissues (8, 152). Because local immunity depends on the stable programming of tissue-adapted cellular states, recurrent exposure to microbial metabolites may reinforce transcriptional and epigenetic programs governing immune homeostasis, tolerance, host defense, tissue repair, and memory (4, 6, 100).

However, microbiota-associated regulation does not require a resident microbial community within every tissue. Whereas metabolites may be produced and sensed locally at densely colonized barrier tissues, effects in distal or low-biomass tissues may instead arise through circulating metabolites, translocated microbial products, or inter-organ immune, neural, and endocrine pathways. Therefore, microbial signatures detected in internal or low-biomass tissues should be interpreted cautiously and distinguished from viable colonization, contamination, transient microbial translocation, or distal exposure to microbiota-derived products.

A concrete framework for future studies should resolve this axis through a series of linked experimental steps. First, the microbial source of a candidate metabolite should be identified using defined microbial consortia, metabolite-deficient bacterial mutants, metagenomics, and metatranscriptomics. Second, stable-isotope tracing combined with longitudinal sampling of intestinal contents, blood, and target tissues should establish metabolite production, transport, tissue penetration, and clearance. Third, spatial metabolomics and cell-type-resolved approaches, including single-cell RNA sequencing, single-cell ATAC sequencing, and CUT&Tag, should identify the responding cell population, altered chromatin mark, and affected gene locus. Fourth, causal involvement should be tested by perturbing the relevant receptor, transporter, metabolic enzyme, or chromatin regulator, followed by metabolite-addition or microbial-restoration experiments. Finally, clearance and secondary-challenge experiments should determine whether the resulting chromatin state is transient or persists as trained immunity, tolerance, or adaptive immune memory.

This experimental framework should be extended to humans using matched stool, blood, and tissue samples collected longitudinally before and after dietary, pharmacological, or microbiota-directed interventions. Integrating microbial composition, metabolite concentrations, chromatin accessibility, transcriptional states, and immune phenotypes within the same individuals would help identify the microbial and host features that predict biological responses. Candidate mechanisms should then be tested in human primary immune cells and tissue-relevant organoid or co-culture systems before clinical evaluation.

Greater methodological standardization will also be necessary. Future studies should report metabolite concentration, route, timing and duration of exposure, diet, medication use, microbial background, tissue distribution, and the activation state of responding cells. In low-biomass tissues, rigorous contamination controls and explicit distinction among viable colonization, microbial DNA or product translocation, and distal metabolite exposure will be essential. Together, these measures would distinguish complete causal pathways from associative observations and improve reproducibility across tissues and experimental models.

Overall, the microbiota–metabolite–epigenome axis provides a framework for understanding how microbial ecology is translated into durable tissue-specific immune states. Establishing where a metabolite originates, how it reaches a target tissue, which cell and chromatin pathway interpret it, and whether the resulting immune program is beneficial or harmful will be necessary to convert this framework into mechanistically defined biomarkers and precisely targeted interventions.

## References

[1] Weitnauer M Mijosek V Dalpke AH . Control of local immunity by airway epithelial cells. Mucosal Immunol. (2016) 9:287–98. doi: 10.1038/mi.2015.126 26627458

[2] De Giovanni M Inverso D Iannacone M . Understanding local immunity to enable regionalized medicine. EMBO J. (2024) 43:5788–92. doi: 10.1038/s44318-024-00255-6 39385044 PMC11612237

[3] Niec RE Rudensky AY Fuchs E . Inflammatory adaptation in barrier tissues. Cell. (2021) 184:3361–75. doi: 10.1016/j.cell.2021.05.036 34171319 PMC8336675

[4] Faria AMC Reis BS Mucida D . Tissue adaptation: Implications for gut immunity and tolerance. J Exp Med. (2017) 214:1211–26. doi: 10.1084/jem.20162014 28432200 PMC5413340

[5] Krausgruber T Fortelny N Fife-Gernedl V Senekowitsch M Schuster LC Lercher A . Structural cells are key regulators of organ-specific immune responses. Nature. (2020) 583:296–302. doi: 10.1038/s41586-020-2424-4 32612232 PMC7610345

[6] Lan Z Chen Z Yang N Liu T Li S Shi Y . Epigenetic control of tissue resident memory T cells. Front Immunol. (2025) 16:1605972. doi: 10.3389/fimmu.2025.1605972 40895552 PMC12394232

[7] Moheimani F Hsu AC Reid AT Williams T Kicic A Stick SM . The genetic and epigenetic landscapes of the epithelium in asthma. Respir Res. (2016) 17:119. doi: 10.1186/s12931-016-0434-4 27658857 PMC5034566

[8] Woo V Alenghat T . Epigenetic regulation by gut microbiota. Gut Microbes. (2022) 14:2022407. doi: 10.1080/19490976.2021.2022407 35000562 PMC8744890

[9] Pepke ML Hansen SB Limborg MT . Unraveling host regulation of gut microbiota through the epigenome-microbiome axis. Trends Microbiol. (2024) 32:1229–40. doi: 10.1016/j.tim.2024.05.006 38839511

[10] Tuong ZK Stewart BJ Guo SA Clatworthy MR . Epigenetics and tissue immunity-Translating environmental cues into functional adaptations. Immunol Rev. (2022) 305:111–36. doi: 10.1111/imr.13036 34821397

[11] Belkaid Y Harrison OJ . Homeostatic immunity and the microbiota. Immunity. (2017) 46:562–76. doi: 10.1016/j.immuni.2017.04.008 28423337 PMC5604871

[12] Ley RE Peterson DA Gordon JI . Ecological and evolutionary forces shaping microbial diversity in the human intestine. Cell. (2006) 124:837–48. doi: 10.1016/j.cell.2006.02.017 16497592

[13] Zheng D Liwinski T Elinav E . Interaction between microbiota and immunity in health and disease. Cell Res. (2020) 30:492–506. doi: 10.1038/s41422-020-0332-7 32433595 PMC7264227

[14] Constantinides MG Belkaid Y . Early-life imprinting of unconventional T cells and tissue homeostasis. Science. (2021) 374:eabf0095. doi: 10.1126/science.abf0095 34882451 PMC8697520

[15] Kouzarides T . Histone acetylases and deacetylases in cell proliferation. Curr Opin Genet Dev. (1999) 9:40–8. doi: 10.1016/s0959-437x(99)80006-9 10072350

[16] Bulusu V Tumanov S Michalopoulou E van den Broek NJ MacKay G Nixon C . Acetate recapturing by nuclear acetyl-coA synthetase 2 prevents loss of histone acetylation during oxygen and serum limitation. Cell Rep. (2017) 18:647–58. doi: 10.1016/j.celrep.2016.12.055 28099844 PMC5276806

[17] Qiu J Villa M Sanin DE Buck MD O'Sullivan D Ching R . Acetate promotes T cell effector function during glucose restriction. Cell Rep. (2019) 27:e5.2063–74. doi: 10.1016/j.celrep.2019.04.022 31091446 PMC6544383

[18] Kaymak I Watson MJ Oswald BM Ma S Johnson BK DeCamp LM . ACLY and ACSS2 link nutrient-dependent chromatin accessibility to CD8 T cell effector responses. J Exp Med. (2024) 221. doi: 10.1084/jem.20231820 39150482 PMC11329787

[19] Li Y Liu A Chen K Li L Zhang X Zou F . Sodium butyrate alleviates lead-induced neuroinflammation and improves cognitive and memory impairment through the ACSS2/H3K9ac/BDNF pathway. Environ Int. (2024) 184:108479. doi: 10.1016/j.envint.2024.108479 38340407

[20] Lund PJ Gates LA Leboeuf M Smith SA Chau L Lopes M . Stable isotope tracing *in vivo* reveals a metabolic bridge linking the microbiota to host histone acetylation. Cell Rep. (2022) 41:111809. doi: 10.1016/j.celrep.2022.111809 36516747 PMC9994635

[21] Puleston DJ Baixauli F Sanin DE Edwards-Hicks J Villa M Kabat AM . Polyamine metabolism is a central determinant of helper T cell lineage fidelity. Cell. (2021) 184:e20.4186–202. doi: 10.1016/j.cell.2021.06.007 34216540 PMC8358979

[22] Porter NJ Christianson DW . Structure, mechanism, and inhibition of the zinc-dependent histone deacetylases. Curr Opin Struct Biol. (2019) 59:9–18. doi: 10.1016/j.sbi.2019.01.004 30743180 PMC6687579

[23] Candido EP Reeves R Davie JR . Sodium butyrate inhibits histone deacetylation in cultured cells. Cell. (1978) 14:105–13. doi: 10.1016/0092-8674(78)90305-7 667927

[24] Shimazu T Hirschey MD Newman J He W Shirakawa K Le Moan N . Suppression of oxidative stress by beta-hydroxybutyrate, an endogenous histone deacetylase inhibitor. Science. (2013) 339:211–4. doi: 10.1126/science.1227166 23223453 PMC3735349

[25] Watson PJ Millard CJ Riley AM Robertson NS Wright LC Godage HY . Insights into the activation mechanism of class I HDAC complexes by inositol phosphates. Nat Commun. (2016) 7:11262. doi: 10.1038/ncomms11262 27109927 PMC4848466

[26] Thomas SP Denu JM . Short-chain fatty acids activate acetyltransferase p300. Elife. (2021) 10. doi: 10.7554/elife.72171 34677127 PMC8585482

[27] Kochumon S Jacob T Koshy M Al-Rashed F Sindhu S Al-Ozairi E . Palmitate Potentiates Lipopolysaccharide-Induced IL-6 Production via Coordinated Acetylation of H3K9/H3K18, p300, and RNA Polymerase II. J Immunol. (2022) 209:731–41. doi: 10.4049/jimmunol.2100928 35896337

[28] Akhter N Kochumon S Hasan A Wilson A Nizam R Al Madhoun A . IFN-gamma and LPS induce synergistic expression of CCL2 in monocytic cells via H3K27 acetylation. J Inflammation Res. (2022) 15:4291–302. doi: 10.2147/jir.s368352 35923906 PMC9343018

[29] Wu Y Wang CZ Wan JY Yao H Yuan CS . Dissecting the interplay mechanism between epigenetics and gut microbiota: health maintenance and disease prevention. Int J Mol Sci. (2021) 22. doi: 10.3390/ijms22136933 34203243 PMC8267743

[30] Sharma M Li Y Stoll ML Tollefsbol TO . The epigenetic connection between the gut microbiome in obesity and diabetes. Front Genet. (2019) 10:1329. doi: 10.3389/fgene.2019.01329 32010189 PMC6974692

[31] Anderson OS Sant KE Dolinoy DC . Nutrition and epigenetics: an interplay of dietary methyl donors, one-carbon metabolism and DNA methylation. J Nutr Biochem. (2012) 23:853–9. doi: 10.1016/j.jnutbio.2012.03.003 22749138 PMC3405985

[32] Mentch SJ Mehrmohamadi M Huang L Liu X Gupta D Mattocks D . Histone methylation dynamics and gene regulation occur through the sensing of one-carbon metabolism. Cell Metab. (2015) 22:861–73. doi: 10.1016/j.cmet.2015.08.024 26411344 PMC4635069

[33] Davison JM Mellott TJ Kovacheva VP Blusztajn JK . Gestational choline supply regulates methylation of histone H3, expression of histone methyltransferases G9a (Kmt1c) and Suv39h1 (Kmt1a), and DNA methylation of their genes in rat fetal liver and brain. J Biol Chem. (2009) 284:1982–9. doi: 10.1074/jbc.m807651200 19001366 PMC2629111

[34] Mehedint MG Niculescu MD Craciunescu CN Zeisel SH . Choline deficiency alters global histone methylation and epigenetic marking at the Re1 site of the calbindin 1 gene. FASEB J. (2010) 24:184–95. doi: 10.1096/fj.09-140145 19752176 PMC2797040

[35] Serefidou M Venkatasubramani AV Imhof A . The impact of one carbon metabolism on histone methylation. Front Genet. (2019) 10:764. doi: 10.3389/fgene.2019.00764 31555321 PMC6722216

[36] Krautkramer KA Dhillon RS Denu JM Carey HV . Metabolic programming of the epigenome: host and gut microbial metabolite interactions with host chromatin. Transl Res. (2017) 189:30–50. doi: 10.1016/j.trsl.2017.08.005 28919341 PMC5659875

[37] Wang L Shannar AAF Wu R Chou P Sarwar MS Kuo HC . Butyrate drives metabolic rewiring and epigenetic reprogramming in human colon cancer cells. Mol Nutr Food Res. (2022) 66:e2200028. doi: 10.1002/mnfr.202200028 35429118 PMC9233026

[38] Ryu TY Kim K Son MY Min JK Kim J Han TS . Downregulation of PRMT1, a histone arginine methyltransferase, by sodium propionate induces cell apoptosis in colon cancer. Oncol Rep. (2019) 41:1691–9. doi: 10.3892/or.2018.6938 30569144 PMC6365698

[39] Lu Y Fan C Liang A Fan X Wang R Li P . Effects of SCFA on the DNA methylation pattern of adiponectin and resistin in high-fat-diet-induced obese male mice. Br J Nutr. (2018) 120:385–92. doi: 10.1017/s0007114518001526 29925443

[40] Fang S Miao J Xiang L Ponugoti B Treuter E Kemper JK . Coordinated recruitment of histone methyltransferase G9a and other chromatin-modifying enzymes in SHP-mediated regulation of hepatic bile acid metabolism. Mol Cell Biol. (2007) 27:1407–24. doi: 10.1128/mcb.00944-06 17145766 PMC1800717

[41] Zhu J B JVdE . AHR and tryptophan metabolism: a collaborative dynamics of immune regulation. Genes Immun. (2024) 25:170–1. doi: 10.1038/s41435-023-00235-6 38104209

[42] Farooqi AA Rakhmetova V Kapanova G Tanbayeva G Mussakhanova A Abdykulova A . Role of ubiquitination and epigenetics in the regulation of ahR signaling in carcinogenesis and metastasis: "Albatross around the neck" or "Blessing in disguise. Cells. (2023) 12. doi: 10.3390/cells12192382 PMC10571945 37830596

[43] Sabari BR Tang Z Huang H Yong-Gonzalez V Molina H Kong HE . Intracellular crotonyl-CoA stimulates transcription through p300-catalyzed histone crotonylation. Mol Cell. (2015) 58:203–15. doi: 10.1016/j.molcel.2015.02.029 25818647 PMC4501262

[44] Fellows R Denizot J Stellato C Cuomo A Jain P Stoyanova E . Microbiota derived short chain fatty acids promote histone crotonylation in the colon through histone deacetylases. Nat Commun. (2018) 9:105. doi: 10.1038/s41467-017-02651-5 29317660 PMC5760624

[45] Hodgkinson K El Abbar F Dobranowski P Manoogian J Butcher J Figeys D . Butyrate's role in human health and the current progress towards its clinical application to treat gastrointestinal disease. Clin Nutr. (2023) 42:61–75. doi: 10.1016/j.clnu.2022.10.024 36502573

[46] Mirzaei R Afaghi A Babakhani S Sohrabi MR Hosseini-Fard SR Babolhavaeji K . Role of microbiota-derived short-chain fatty acids in cancer development and prevention. BioMed Pharmacother. (2021) 139:111619. doi: 10.1016/j.biopha.2021.111619 33906079

[47] Nikolaieva N Sevcikova A Omelka R Martiniakova M Mego M Ciernikova S . Gut microbiota-microRNA interactions in intestinal homeostasis and cancer development. Microorganisms. (2022) 11. doi: 10.3390/microorganisms11010107 36677399 PMC9867529

[48] Godman CA Joshi R Tierney BR Greenspan E Rasmussen TP Wang HW . HDAC3 impacts multiple oncogenic pathways in colon cancer cells with effects on Wnt and vitamin D signaling. Cancer Biol Ther. (2008) 7:1570–80. doi: 10.4161/cbt.7.10.6561 18769117 PMC2614677

[49] Shaffer AL Lin KI Kuo TC Yu X Hurt EM Rosenwald A . Blimp-1 orchestrates plasma cell differentiation by extinguishing the mature B cell gene expression program. Immunity. (2002) 17:51–62. doi: 10.1016/s1074-7613(02)00335-7 12150891

[50] White CA Pone EJ Lam T Tat C Hayama KL Li G . Histone deacetylase inhibitors upregulate B cell microRNAs that silence AID and Blimp-1 expression for epigenetic modulation of antibody and autoantibody responses. J Immunol. (2014) 193:5933–50. doi: 10.4049/jimmunol.1401702 25392531 PMC4258531

[51] Yadav DK Shrestha S Lillycrop KA Joglekar CV Pan H Holbrook JD . Vitamin B(12) supplementation influences methylation of genes associated with type 2 diabetes and its intermediate traits. Epigenomics. (2018) 10:71–90. doi: 10.2217/epi-2017-0102 29135286

[52] Epelman S Lavine KJ Randolph GJ . Origin and functions of tissue macrophages. Immunity. (2014) 41:21–35. doi: 10.1016/j.immuni.2014.06.013 25035951 PMC4470379

[53] Lavin Y Winter D Blecher-Gonen R David E Keren-Shaul H Merad M . Tissue-resident macrophage enhancer landscapes are shaped by the local microenvironment. Cell. (2014) 159:1312–26. doi: 10.1016/j.cell.2014.11.018 25480296 PMC4437213

[54] Sarkar A Mitra P Lahiri A Das T Sarkar J Paul S . Butyrate limits inflammatory macrophage niche in NASH. Cell Death Dis. (2023) 14:332. doi: 10.1038/s41419-023-05853-6 37202387 PMC10195803

[55] Ji J Shu D Zheng M Wang J Luo C Wang Y . Microbial metabolite butyrate facilitates M2 macrophage polarization and function. Sci Rep. (2016) 6:24838. doi: 10.1038/srep24838 27094081 PMC4837405

[56] Song S Shi K Fan M Wen X Li J Guo Y . Clostridium butyricum and its metabolites regulate macrophage polarization through miR-146a to antagonize gouty arthritis. J Adv Res. (2026) 80:943–60. doi: 10.1016/j.jare.2025.05.036 40398744 PMC12869238

[57] Zhang L Ma Z Zhang X Wang J Tian W Ren Y . Butyrate alleviates alcoholic liver disease-associated inflammation through macrophage regulation and polarization via the HDAC1/miR-155 axis. Int Immunopharmacol. (2024) 131:111852. doi: 10.1016/j.intimp.2024.111852 38492338

[58] Chang PV Hao L Offermanns S Medzhitov R . The microbial metabolite butyrate regulates intestinal macrophage function via histone deacetylase inhibition. Proc Natl Acad Sci USA. (2014) 111:2247–52. doi: 10.1073/pnas.1322269111 24390544 PMC3926023

[59] Park JW Kim HY Kim MG Jeong S Yun CH Han SH . Short-chain fatty acids inhibit staphylococcal lipoprotein-induced nitric oxide production in murine macrophages. Immune Netw. (2019) 19:e9. doi: 10.4110/in.2019.19.e9 31089436 PMC6494764

[60] Bahman F Kochumon S Malik MZ Akther N Jacob S Drobiova H . H3K9 acetylation-NF-kappaB-AP-1 nexus targeted by ITE limits TNF-alpha-induced MMP-9 expression in monocytic cells. J Immunol. (2026) 215. doi: 10.1093/jimmun/vkaf240 41014589

[61] Schulthess J Pandey S Capitani M Rue-Albrecht KC Arnold I Franchini F . The short chain fatty acid butyrate imprints an antimicrobial program in macrophages. Immunity. (2019) 50:e7.432–45. doi: 10.1016/j.immuni.2018.12.018 30683619 PMC6382411

[62] Li N Gong Y Zhu Y Li B Wang C Wang Z . Exogenous acetate attenuates inflammatory responses through HIF-1alpha-dependent glycolysis regulation in macrophage. Cell Mol Life Sci. (2024) 82:21. doi: 10.1007/s00018-024-05521-8 39725781 PMC11671453

[63] Chemudupati M Kenney AD Smith AC Fillinger RJ Zhang L Zani A . Butyrate reprograms expression of specific interferon-stimulated genes. J Virol. (2020) 94. doi: 10.1128/jvi.00326-20 32461320 PMC7394905

[64] Chen K Wang JM Yuan R Yi X Li L Gong W . Tissue-resident dendritic cells and diseases involving dendritic cell malfunction. Int Immunopharmacol. (2016) 34:1–15. doi: 10.1016/j.intimp.2016.02.007 26906720 PMC4818737

[65] Boukhaled GM Corrado M Guak H Krawczyk CM . Chromatin architecture as an essential determinant of dendritic cell function. Front Immunol. (2019) 10:1119. doi: 10.3389/fimmu.2019.01119 31214161 PMC6557980

[66] Wang M Su H Liu J . Metabolic crosstalk between intestinal microbiota and dendritic cells: from homeostasis to inflammation. Acta Biochim Biophys Sin (Shanghai). (2025) 58:156–68. doi: 10.3724/abbs.2025231 41408830 PMC12862611

[67] Singh N Thangaraju M Prasad PD Martin PM Lambert NA Boettger T . Blockade of dendritic cell development by bacterial fermentation products butyrate and propionate through a transporter (Slc5a8)-dependent inhibition of histone deacetylases. J Biol Chem. (2010) 285:27601–8. doi: 10.1074/jbc.m110.102947 20601425 PMC2934627

[68] Leon B . Type 2 conventional dendritic cell functional heterogeneity: ontogenically committed or environmentally plastic? Trends Immunol. (2025) 46:104–20. doi: 10.1016/j.it.2024.12.005 PMC11835539 39843310

[69] Andrusaite A Lewis J Frede A Farthing A Kastele V Montgomery J . Microbiota-derived butyrate inhibits cDC development via HDAC inhibition, diminishing their ability to prime T cells. Mucosal Immunol. (2024) 17:1199–211. doi: 10.1016/j.mucimm.2024.08.003 39142634 PMC11631772

[70] Liu L Li L Min J Wang J Wu H Zeng Y . Butyrate interferes with the differentiation and function of human monocyte-derived dendritic cells. Cell Immunol. (2012) 277:66–73. doi: 10.1016/j.cellimm.2012.05.011 22698927

[71] Millard AL Mertes PM Ittelet D Villard F Jeannesson P Bernard J . Butyrate affects differentiation, maturation and function of human monocyte-derived dendritic cells and macrophages. Clin Exp Immunol. (2002) 130:245–55. doi: 10.1046/j.0009-9104.2002.01977.x 12390312 PMC1906513

[72] Wang B Morinobu A Horiuchi M Liu J Kumagai S . Butyrate inhibits functional differentiation of human monocyte-derived dendritic cells. Cell Immunol. (2008) 253:54–8. doi: 10.1016/j.cellimm.2008.04.016 18522857

[73] Kaisar MMM Pelgrom LR van der Ham AJ Yazdanbakhsh M Everts B . Butyrate conditions human dendritic cells to prime type 1 regulatory T cells via both histone deacetylase inhibition and G protein-coupled receptor 109A signaling. Front Immunol. (2017) 8:1429. doi: 10.3389/fimmu.2017.01429 29163504 PMC5670331

[74] Tang Q Fan G Peng X Sun X Kong X Zhang L . Gut bacterial L-lysine alters metabolism and histone methylation to drive dendritic cell tolerance. Cell Rep. (2025) 44:115125. doi: 10.1016/j.celrep.2024.115125 39932193

[75] Inamoto T Furuta K Han C Uneme M Kano T Ishikawa K . Short-chain fatty acids stimulate dendrite elongation in dendritic cells by inhibiting histone deacetylase. FEBS J. (2023) 290:5794–810. doi: 10.1111/febs.16945 37646105

[76] Zhang Q Zhao Q Li T Lu L Wang F Zhang H . Lactobacillus plantarum-derived indole-3-lactic acid ameliorates colorectal tumorigenesis via epigenetic regulation of CD8(+) T cell immunity. Cell Metab. (2023) 35:e9.943–60. doi: 10.1016/j.cmet.2023.04.015 37192617

[77] Devaux CA Raoult D . The microbiological memory, an epigenetic regulator governing the balance between good health and metabolic disorders. Front Microbiol. (2018) 9:1379. doi: 10.3389/fmicb.2018.01379 29997595 PMC6028609

[78] Luu M Weigand K Wedi F Breidenbend C Leister H Pautz S . Regulation of the effector function of CD8(+) T cells by gut microbiota-derived metabolite butyrate. Sci Rep. (2018) 8:14430. doi: 10.1038/s41598-018-32860-x 30258117 PMC6158259

[79] Feng Q Liu Z Yu X Huang T Chen J Wang J . Lactate increases stemness of CD8 + T cells to augment anti-tumor immunity. Nat Commun. (2022) 13:4981. doi: 10.1038/s41467-022-32521-8 36068198 PMC9448806

[80] Bachem A Makhlouf C Binger KJ de Souza DP Tull D Hochheiser K . Microbiota-derived short-chain fatty acids promote the memory potential of antigen-activated CD8(+) T cells. Immunity. (2019) 51:e5.285–97. doi: 10.1016/j.immuni.2019.06.002 31272808

[81] Jia D Wang Q Qi Y Jiang Y He J Lin Y . Microbial metabolite enhances immunotherapy efficacy by modulating T cell stemness in pan-cancer. Cell. (2024) 187:e21.1651–65. doi: 10.1016/j.cell.2024.02.022 38490195

[82] Luo A Leach ST Barres R Hesson LB Grimm MC Simar D . The microbiota and epigenetic regulation of T helper 17/regulatory T cells: in search of a balanced immune system. Front Immunol. (2017) 8:417. doi: 10.3389/fimmu.2017.00417 28443096 PMC5385369

[83] Luu M Pautz S Kohl V Singh R Romero R Lucas S . The short-chain fatty acid pentanoate suppresses autoimmunity by modulating the metabolic-epigenetic crosstalk in lymphocytes. Nat Commun. (2019) 10:760. doi: 10.1038/s41467-019-08711-2 30770822 PMC6377655

[84] Chen X Sunkel B Wang M Kang S Wang T Gnanaprakasam JNR . Succinate dehydrogenase/complex II is critical for metabolic and epigenetic regulation of T cell proliferation and inflammation. Sci Immunol. (2022) 7:eabm8161. doi: 10.1126/sciimmunol.abm8161 35486677 PMC9332111

[85] Sanchez HN Moroney JB Gan H Shen T Im JL Li T . B cell-intrinsic epigenetic modulation of antibody responses by dietary fiber-derived short-chain fatty acids. Nat Commun. (2020) 11:60. doi: 10.1038/s41467-019-13603-6 31896754 PMC6940392

[86] Vivier E Artis D Colonna M Diefenbach A Di Santo JP Eberl G . Innate lymphoid cells: 10 years on. Cell. (2018) 174:1054–66. doi: 10.1016/j.cell.2018.07.017 30142344

[87] Meininger I Carrasco A Rao A Soini T Kokkinou E Mjosberg J . Tissue-specific features of innate lymphoid cells. Trends Immunol. (2020) 41:902–17. doi: 10.1016/j.it.2020.08.009 32917510

[88] Schneider C O'Leary CE von Moltke J Liang HE Ang QY Turnbaugh PJ . A metabolite-triggered tuft cell-ILC2 circuit drives small intestinal remodeling. Cell. (2018) 174:e14.271–84. doi: 10.1016/j.cell.2018.05.014 29887373 PMC6046262

[89] Martinez-Gonzalez I Takei F . New insights into ILC2 memory. Immunol Rev. (2024) 323:118–25. doi: 10.1111/imr.13323 38506432

[90] Zhang X Gao X Liu Z Shao F Yu D Zhao M . Microbiota regulates the TET1-mediated DNA hydroxymethylation program in innate lymphoid cell differentiation. Nat Commun. (2024) 15:4792. doi: 10.1038/s41467-024-48794-0 38839760 PMC11153590

[91] Thio CL Chi PY Lai AC Chang YJ . Regulation of type 2 innate lymphoid cell-dependent airway hyperreactivity by butyrate. J Allergy Clin Immunol. (2018) 142:e12.1867–83. doi: 10.1016/j.jaci.2018.02.032 29522844

[92] Jiang M Wang J Li Z Xu D Jing J Li F . Dietary fiber-derived microbial butyrate suppresses ILC2-dependent airway inflammation in COPD. Mediators Inflammation. (2024) 2024:6263447. doi: 10.1155/2024/6263447 39015676 PMC11251798

[93] Yang W Yu T Huang X Bilotta AJ Xu L Lu Y . Intestinal microbiota-derived short-chain fatty acids regulation of immune cell IL-22 production and gut immunity. Nat Commun. (2020) 11:4457. doi: 10.1038/s41467-020-18262-6 32901017 PMC7478978

[94] Li S Bostick JW Ye J Qiu J Zhang B Urban JF Jr. . Aryl hydrocarbon receptor signaling cell intrinsically inhibits intestinal group 2 innate lymphoid cell function. Immunity. (2018) 49:e5.915–28. doi: 10.1016/j.immuni.2018.09.015 30446384 PMC6249058

[95] Li Y Liu N Ge Y Yang Y Ren F Wu Z . Tryptophan and the innate intestinal immunity: crosstalk between metabolites, host innate immune cells, and microbiota. Eur J Immunol. (2022) 52:856–68. doi: 10.1002/eji.202149401 35362153

[96] Netea MG Dominguez-Andres J Barreiro LB Chavakis T Divangahi M Fuchs E . Defining trained immunity and its role in health and disease. Nat Rev Immunol. (2020) 20:375–88. doi: 10.1038/s41577-020-0285-6 32132681 PMC7186935

[97] Quintin J Saeed S Martens JHA Giamarellos-Bourboulis EJ Ifrim DC Logie C . Candida albicans infection affords protection against reinfection via functional reprogramming of monocytes. Cell Host Microbe. (2012) 12:223–32. doi: 10.1016/j.chom.2012.06.006 22901542 PMC3864037

[98] Arts RJ Novakovic B Ter Horst R Carvalho A Bekkering S Lachmandas E . Glutaminolysis and fumarate accumulation integrate immunometabolic and epigenetic programs in trained immunity. Cell Metab. (2016) 24:807–18. doi: 10.1016/j.cmet.2016.10.008 27866838 PMC5742541

[99] Kim HY Kang YJ Kim DH Jang J Lee SJ Kim G . Uremic toxin indoxyl sulfate induces trained immunity via the AhR-dependent arachidonic acid pathway in end-stage renal disease (ESRD). Elife. (2024) 12. doi: 10.7554/elife.87316.3 PMC11233136 38980302

[100] Buquicchio FA Fonseca R Yan PK Wang F Evrard M Obers A . Distinct epigenomic landscapes underlie tissue-specific memory T cell differentiation. Immunity. (2024) 57:e6.2202–15. doi: 10.1016/j.immuni.2024.06.014 39043184

[101] Hooper LV Macpherson AJ . Immune adaptations that maintain homeostasis with the intestinal microbiota. Nat Rev Immunol. (2010) 10:159–69. doi: 10.1038/nri2710 20182457

[102] Furusawa Y Obata Y Fukuda S Endo TA Nakato G Takahashi D . Commensal microbe-derived butyrate induces the differentiation of colonic regulatory T cells. Nature. (2013) 504:446–50. doi: 10.1038/nature12721 24226770

[103] Arpaia N Campbell C Fan X Dikiy S van der Veeken J deRoos P . Metabolites produced by commensal bacteria promote peripheral regulatory T-cell generation. Nature. (2013) 504:451–5. doi: 10.1038/nature12726 24226773 PMC3869884

[104] Smith PM Howitt MR Panikov N Michaud M Gallini CA Bohlooly YM . The microbial metabolites, short-chain fatty acids, regulate colonic Treg cell homeostasis. Science. (2013) 341:569–73. doi: 10.1126/science.1241165 23828891 PMC3807819

[105] Li W Hang S Fang Y Bae S Zhang Y Zhang M . A bacterial bile acid metabolite modulates T(reg) activity through the nuclear hormone receptor NR4A1. Cell Host Microbe. (2021) 29:e9.1366–77. doi: 10.1016/j.chom.2021.07.013 34416161 PMC9064000

[106] Li R Li J Zhou X . Lung microbiome: new insights into the pathogenesis of respiratory diseases. Signal Transduct Target Ther. (2024) 9:19. doi: 10.1038/s41392-023-01722-y 38228603 PMC10791971

[107] Trompette A Gollwitzer ES Yadava K Sichelstiel AK Sprenger N Ngom-Bru C . Gut microbiota metabolism of dietary fiber influences allergic airway disease and hematopoiesis. Nat Med. (2014) 20:159–66. doi: 10.1038/nm.3444 24390308

[108] Liu X Yang C Hu Y Lei E Lin X Zhao L . HIST1H1C regulates interferon-beta and inhibits influenza virus replication by interacting with IRF3. Front Immunol. (2017) 8:350. doi: 10.3389/fimmu.2017.00350 28392790 PMC5364133

[109] Anastasakis DG Benhalevy D Cuburu N Altan-Bonnet N Hafner M . Epigenetic repression of antiviral genes by SARS-CoV-2 NSP1. PLoS One. (2024) 19:e0297262. doi: 10.1371/journal.pone.0297262 38277395 PMC10817131

[110] Kee J Thudium S Renner DM Glastad K Palozola K Zhang Z . SARS-CoV-2 disrupts host epigenetic regulation via histone mimicry. Nature. (2022) 610:381–8. doi: 10.1038/s41586-022-05282-z 36198800 PMC9533993

[111] Feng Q Su Z Song S Chiu H Zhang B Yi L . Histone deacetylase inhibitors suppress RSV infection and alleviate virus-induced airway inflammation. Int J Mol Med. (2016) 38:812–22. doi: 10.3892/ijmm.2016.2691 27460781 PMC4990302

[112] Marcos-Villar L Nistal-Villan E Zamarreno N Garaigorta U Gastaminza P Nieto A . Interferon-β stimulation elicited by the influenza virus is regulated by the histone methylase Dot1L through the RIG-I-TRIM25 signaling axis. Cells. (2020) 9. doi: 10.3390/cells9030732 32188146 PMC7140698

[113] Marrella V Nicchiotti F Cassani B . Microbiota and immunity during respiratory infections: Lung and gut affair. Int J Mol Sci. (2024) 25. doi: 10.3390/ijms25074051 38612860 PMC11012346

[114] Whiteside SA McGinniss JE Collman RG . The lung microbiome: progress and promise. J Clin Invest. (2021) 131. doi: 10.1172/jci150473 34338230 PMC8321564

[115] Sanford JA Zhang LJ Williams MR Gangoiti JA Huang CM Gallo RL . Inhibition of HDAC8 and HDAC9 by microbial short-chain fatty acids breaks immune tolerance of the epidermis to TLR ligands. Sci Immunol. (2016) 1. doi: 10.1126/sciimmunol.aah4609 28783689

[116] Sanford JA O'Neill AM Zouboulis CC Gallo RL . Short-chain fatty acids from Cutibacterium acnes activate both a canonical and epigenetic inflammatory response in human sebocytes. J Immunol. (2019) 202:1767–76. doi: 10.4049/jimmunol.1800893 30737272 PMC7251550

[117] Harrison OJ Linehan JL Shih HY Bouladoux N Han SJ Smelkinson M . Commensal-specific T cell plasticity promotes rapid tissue adaptation to injury. Science. (2019) 363. doi: 10.1126/science.aat6280 30523076 PMC7304459

[118] Qiu Z Zhu Z Liu X Chen B Yin H Gu C . A dysregulated sebum-microbial metabolite-IL-33 axis initiates skin inflammation in atopic dermatitis. J Exp Med. (2022) 219. doi: 10.1084/jem.20212397 35977109 PMC9375142

[119] Luo CH Lai AC Chang YJ . Butyrate inhibits Staphylococcus aureus-aggravated dermal IL-33 expression and skin inflammation through histone deacetylase inhibition. Front Immunol. (2023) 14:1114699. doi: 10.3389/fimmu.2023.1114699 37261337 PMC10228744

[120] Li H Gao Z Zhang J Ye X Xu A Ye J . Sodium butyrate stimulates expression of fibroblast growth factor 21 in liver by inhibition of histone deacetylase 3. Diabetes. (2012) 61:797–806. doi: 10.2337/db11-0846 22338096 PMC3314370

[121] Luo M Du Y Liu X Zhang S Zhu W Liu K . Fecal microbiota transplantation alleviates cirrhotic portal hypertension in rats via butyrate-mediated HDAC3 inhibition and PI3K/Akt/eNOS signaling regulation. Eur J Pharmacol. (2025) 1002:177781. doi: 10.1016/j.ejphar.2025.177781 40441587

[122] Saiman Y Shen TD Lund PJ Gershuni VM Jang C Patel S . Global microbiota-dependent histone acetylation patterns are irreversible and independent of short chain fatty acids. Hepatology. (2021) 74:3427–40. doi: 10.1002/hep.32043 34233020 PMC9867598

[123] Qiao YL Qian JM Wang FR Ma ZY Wang QW . Butyrate protects liver against ischemia reperfusion injury by inhibiting nuclear factor kappa B activation in Kupffer cells. J Surg Res. (2014) 187:653–9. doi: 10.1016/j.jss.2013.08.028 24445056

[124] Sun J Wu Q Sun H Qiao Y . Inhibition of histone deacetylase by butyrate protects rat liver from ischemic reperfusion injury. Int J Mol Sci. (2014) 15:21069–79. doi: 10.3390/ijms151121069 25405737 PMC4264212

[125] Kamm DR McCommis KS . Hepatic stellate cells in physiology and pathology. J Physiol. (2022) 600:1825–37. doi: 10.1113/jp281061 35307840 PMC9012702

[126] Niki T Rombouts K De Bleser P De Smet K Rogiers V Schuppan D . A histone deacetylase inhibitor, trichostatin A, suppresses myofibroblastic differentiation of rat hepatic stellate cells in primary culture. Hepatology. (1999) 29:858–67. doi: 10.1002/hep.510290328 10051490

[127] Pirola CJ Salatino A Fernandez Gianotti T Castano GO Garaycoechea M Sookoian S . Cross talk between the liver microbiome and epigenome in patients with metabolic dysfunction-associated steatotic liver disease. EBioMedicine. (2024) 101:104996. doi: 10.1016/j.ebiom.2024.104996 38320344 PMC10862506

[128] Louveau A Harris TH Kipnis J . Revisiting the mechanisms of CNS immune privilege. Trends Immunol. (2015) 36:569–77. doi: 10.1016/j.it.2015.08.006 26431936 PMC4593064

[129] Louveau A Smirnov I Keyes TJ Eccles JD Rouhani SJ Peske JD . Structural and functional features of central nervous system lymphatic vessels. Nature. (2015) 523:337–41. doi: 10.1038/nature14432 26030524 PMC4506234

[130] Tremblay ME Stevens B Sierra A Wake H Bessis A Nimmerjahn A . The role of microglia in the healthy brain. J Neurosci. (2011) 31:16064–9. doi: 10.1523/JNEUROSCI.4158-11.2011 PMC6633221 22072657

[131] Erny D Hrabe de Angelis AL Jaitin D Wieghofer P Staszewski O David E . Host microbiota constantly control maturation and function of microglia in the CNS. Nat Neurosci. (2015) 18:965–77. doi: 10.1038/nn.4030 26030851 PMC5528863

[132] Rutsch A Kantsjo JB Ronchi F . The gut-brain axis: How microbiota and host inflammasome influence brain physiology and pathology. Front Immunol. (2020) 11:604179. doi: 10.3389/fimmu.2020.604179 33362788 PMC7758428

[133] Loh JS Mak WQ Tan LKS Ng CX Chan HH Yeow SH . Microbiota-gut-brain axis and its therapeutic applications in neurodegenerative diseases. Signal Transduct Target Ther. (2024) 9:37. doi: 10.1038/s41392-024-01743-1 38360862 PMC10869798

[134] Bajinka O Simbilyabo L Tan Y Jabang J Saleem SA . Lung-brain axis. Crit Rev Microbiol. (2022) 48:257–69. doi: 10.1080/1040841x.2021.1960483 34348558

[135] Erny D Dokalis N Mezo C Castoldi A Mossad O Staszewski O . Microbiota-derived acetate enables the metabolic fitness of the brain innate immune system during health and disease. Cell Metab. (2021) 33:e7.2260–76. doi: 10.1016/j.cmet.2021.10.010 34731656

[136] Patnala R Arumugam TV Gupta N Dheen ST . HDAC inhibitor sodium butyrate-mediated epigenetic regulation enhances neuroprotective function of microglia during ischemic stroke. Mol Neurobiol. (2017) 54:6391–411. doi: 10.1007/s12035-016-0149-z 27722928

[137] Subramanian A Gupta A Saxena S Gupta A Kumar R Nigam A . Proton MR CSF analysis and a new software as predictors for the differentiation of meningitis in children. NMR BioMed. (2005) 18:213–25. doi: 10.1002/nbm.944 15627241

[138] Vijay N Morris ME . Role of monocarboxylate transporters in drug delivery to the brain. Curr Pharm Des. (2014) 20:1487–98. doi: 10.2174/13816128113199990462 23789956 PMC4084603

[139] Williams-Karnesky RL Sandau US Lusardi TA Lytle NK Farrell JM Pritchard EM . Epigenetic changes induced by adenosine augmentation therapy prevent epileptogenesis. J Clin Invest. (2013) 123:3552–59. doi: 10.1172/jci65636 23863710 PMC3726154

[140] Eshleman EM Rice T Potter C Waddell A Hashimoto-Hill S Woo V . Microbiota-derived butyrate restricts tuft cell differentiation via histone deacetylase 3 to modulate intestinal type 2 immunity. Immunity. (2024) 57:e6.319–32. doi: 10.1016/j.immuni.2024.01.002 38295798 PMC10901458

[141] Liu Z Zhang Q Zhang H Yi Z Ma H Wang X . Colorectal cancer microbiome programs DNA methylation of host cells by affecting methyl donor metabolism. Genome Med. (2024) 16:77. doi: 10.1186/s13073-024-01344-1 38840170 PMC11151592

[142] Mann ER Lam YK Uhlig HH . Short-chain fatty acids: linking diet, the microbiome and immunity. Nat Rev Immunol. (2024) 24:577–95. doi: 10.1038/s41577-024-01014-8 38565643

[143] Hugenholtz F de Vos WM . Mouse models for human intestinal microbiota research: a critical evaluation. Cell Mol Life Sci. (2018) 75:149–60. doi: 10.1007/s00018-017-2693-8 29124307 PMC5752736

[144] Park JC Im SH . Of men in mice: the development and application of a humanized gnotobiotic mouse model for microbiome therapeutics. Exp Mol Med. (2020) 52:1383–96. doi: 10.1038/s12276-020-0473-2 32908211 PMC8080820

[145] van der Beek CM Canfora EE Kip AM Gorissen SHM Olde Damink SWM van Eijk HM . The prebiotic inulin improves substrate metabolism and promotes short-chain fatty acid production in overweight to obese men. Metabolism. (2018) 87:25–35. doi: 10.1016/j.metabol.2018.06.009 29953876

[146] Canale FP Basso C Antonini G Perotti M Li N Sokolovska A . Metabolic modulation of tumours with engineered bacteria for immunotherapy. Nature. (2021) 598:662–6. doi: 10.1038/s41586-021-04003-2 34616044

[147] Seekatz AM Theriot CM Rao K Chang YM Freeman AE Kao JY . Restoration of short chain fatty acid and bile acid metabolism following fecal microbiota transplantation in patients with recurrent Clostridium difficile infection. Anaerobe. (2018) 53:64–73. doi: 10.1016/j.anaerobe.2018.04.001 29654837 PMC6185828

[148] Fan X Zhang Z Gao W Pan Q Luo K He B . An engineered butyrate-derived polymer nanoplatform as a mucosa-healing enhancer potentiates the therapeutic effect of magnolol in inflammatory bowel disease. ACS Nano. (2024) 18:229–44. doi: 10.1021/acsnano.3c05732 38112525

[149] Kespohl M Vachharajani N Luu M Harb H Pautz S Wolff S . The microbial metabolite butyrate induces expression of Th1-associated factors in CD4(+) T cells. Front Immunol. (2017) 8:1036. doi: 10.3389/fimmu.2017.01036 28894447 PMC5581317

[150] Yang K Hou Y Zhang Y Liang H Sharma A Zheng W . Suppression of local type I interferon by gut microbiota-derived butyrate impairs antitumor effects of ionizing radiation. J Exp Med. (2021) 218. doi: 10.1084/jem.20201915 33496784 PMC7844434

[151] Nastasi C Fredholm S Willerslev-Olsen A Hansen M Bonefeld CM Geisler C . Butyrate and propionate inhibit antigen-specific CD8(+) T cell activation by suppressing IL-12 production by antigen-presenting cells. Sci Rep. (2017) 7:14516. doi: 10.1038/s41598-017-15099-w 29109552 PMC5673935

[152] Kim CH . Immune regulation by microbiome metabolites. Immunology. (2018) 154:220–9. doi: 10.1111/imm.12930 29569377 PMC5980225

[153] Ngo VL Lieber CM Abo H Kuczma M Zou J Plemper RK . Segmented filamentous bacteria reprogramming of alveolar macrophages limits postinfluenza bacterial pneumonia. Sci Immunol. (2026) 11:eadt8858. doi: 10.1126/sciimmunol.adt8858 41481698 PMC13383210

